# Supplementary material for: A monoacylglycerol lipase inhibitor showing therapeutic efficacy in mice without central side effects or dependence
Source: Nat Commun. 2023 Dec 5;14:8039. doi: 10.1038/s41467-023-43606-3 (PMC10698032; doi:10.1038/s41467-023-43606-3)

SUPPORTING INFORMATION
for

A monoacylglycerol lipase inhibitor showing therapeutic efficacy in mice without central side effects or dependence

Ming Jiang[1]†, Mirjam C. W. Huizenga[1]†, Jonah L. Wirt[2]†, Janos Paloczi[3], Avand Amedi[1], Richard J. B. H. N. van der Berg[4], Joerg Benz[5], Ludovic Collin[5], Hui Deng[1], Xinyu Di[6], Wouter F. Driever[1], Bogdan F. Florea[4], Uwe Grether[5], Antonius P. A. Janssen[1], Thomas Hankemeier[6], Laura H. Heitman[7], Tsang-Wai Lam[8], Florian Mohr[1], Anto Pavlovic[5], Iris Ruf[5], Helma van den Hurk[8], Anna F. Stevens[1], Daan van der Vliet[1], Tom van der Wel[1], Matthias B. Wittwer[5], Constant A.A. van Boeckel[1], Pal Pacher[3], Andrea G. Hohmann[2*], Mario van der Stelt[1*]

[1]Department of Molecular Physiology, Leiden University & Oncode Institute, Netherlands; [2]Department of Psychological and Brain Sciences, Program in Neuroscience, Gill Center for Biomolecular Science, Indiana University, Bloomington, IN, USA; [3]Laboratory of Cardiovascular Physiology and Tissue Injury, National Institute of Health/NIAAA, Rockville, Maryland, USA; [4]Department of Bio-organic Synthesis, Leiden University, Netherlands; [5]Roche Innovation Center Basel, F. Hoffmann-La Roche Ltd., Basel, Switzerland; [6]Metabolomics and analytics center, Leiden University, Netherlands; [7]Division of Drug Discovery and Safety, Leiden University & Oncode Institute, Netherlands; [8]Pivot Park Screening Centre, Oss, Netherlands

*Corresponding authors: m.van.der.stelt@chem.leidenuniv.nl; hohmanna@indiana.edu

†These authors contributed equally to this work

Supplementary Table 1 – High-throughput screening (HTS) data of the hMAGL activity assay.

Category	Parameter	Description
Assay	Type of assay	*In vitro* enzyme fluorescence-based activity assay
	Target	MAGL (human)
	Primary measurement	MAGL-mediated hydrolysis of 2-arachidonoylglycerol (2-AG), coupled to an enzymatic cascade reaction that ultimately converts Amplex™ Red into fluorescent resorufin.
	Key reagents	Membrane fractions of hMAGL-FLAG-overexpressing HEK293T cells; 2-AG (Cayman); glycerol kinase (GK); glycerol-3-phosphate oxidase (GPO); horseradish peroxidase (HRP); ATP; Amplex™ Red (all Sigma Aldrich).
	Assay protocol	1x Assay buffer (50 mM HEPES pH 7.4, 1 mM EDTA, 5 mM $MgCl_2$, 100 mM NaCl, 0.5% (w/w) BSA, 0.03% (w/w) Tween-20.<br><br>Method:<br>1. Add 20 nL of compound (final concentration 10 µM, final DMSO concentration 0.5%)<br>2. Add 20 nL of DMSO to negative control (min) wells or 20 nL of JZL184 to positive control (max) wells (final concentration 20 µM; 0.5% DMSO).<br>3. Add 1 µL of assay buffer to all wells.<br>4. Add 2 µL of hMAGL membranes in assay buffer (final concentration 9 ng/µL).<br>5. Incubate for 30 min at rt.<br>6. Add 1 µL of substrate mix in assay buffer (final concentrations 0.2 U/mL GK, GPO and HRP, 125 µM ATP, 10 µM Amplex™ Red, 25 µM 2-AG).<br>7. Incubate for 45 min at rt in the dark.<br>8. Read fluorescence intensity (excitation 531 nm, emission 595 nm) on Envision plate reader.
Library	Library size	233,820 compounds
	Source	Cancer Drug Discovery Initiative (https://cddi.nl), performed at Pivot Park Screening Centre B.V. in Oss, The Netherlands.
Screen	Format	Black 1536-well plate (Corning #3724)
	Concentration(s) tested	10 µM, final DMSO concentration 0.5%
	Plate controls	DMSO for the minimum effect (0% inhibition), 20 µM JZL184 for the maximum effect (100% inhibition).
	Dispensing system	Echo-555 for dispensing DMSO or compounds; BioRAPTR for dispensing<br>1 µL assay buffer; Multidrop-Combi for dispensing 2 µL hMAGL-membranes; Certus for dispensing 1 µL substrate mix.
	Detection instrument	Envision reader (Perkin Elmer)
	Assay validation / QC	Over 188 plates, the Z'-factor varied from 0.61 to 0.90 and the S/B-ratio from 4.16 to 10.25.
Post-HTS analysis	Hit criteria	Z-score < -4.95 (z-score = X - μ/σ) where X = measured effect (residual activity), μ = mean effect, σ = standard deviation.
	Hit rate	0.67% (1,555 compounds with ≤ 50% residual activity).

Additional assay(s)	The primary assay identified 1,555 active hits, which were expanded to 4,389 compounds using a nearest neighbor clustering algorithm. These compounds were then tested again in the primary assay (confirmation assay), resulting in 1,142 confirmed actives. As a deselection assay, the primary assay was repeated but with 12.5 μM glycerol replacing 2-AG as the substrate. Deselection (< 10% inhibition in deselection assay) resulted in 334 remaining compounds. Using a more stringent cutoff (> 60% inhibition in primary assay), this number was reduced to 111 compounds. A triaging process, involved examination of intellectual property and an apparent reversible mode of action, judged by the absence of structural motifs that are commonly present in irreversible serine hydrolase inhibitors, such as lactones and activated ureas and carbamates. The remaining 50 actives were then measured in dose-response experiments (7 equidistant concentration steps from 10 μM to 10 nM) in the primary assay.
Confirmation of hit purity and structure	Compounds were checked for correct MW and purity (>90%) by LC-MS analysis.

Supplementary Table 2 – Qualified hit list. Hits are clustered by chemotype. Purity (> 90%) and mass were confirmed by LC-MS. Deviations from expected mass are shown as ΔMW. pIC_{50} values were determined in end point measurement after 45 min. Percentages inhibition in orthogonal ABPP assay are relative to vehicle-treated controls. Physicochemical properties (cLogP, tPSA, HBD, HBA) were calculated using ChemDraw Professional 16.0. MW: molecular weight; tPSA: topological polar surface area; HBD: number of hydrogen bond donors; HBA: number of hydrogen bond acceptors; LipE: lipophilic efficiency (LipE = pIC_{50} primary assay – cLogP); LE: ligand efficiency (LE = 1.4 x pIC_{50}/N where N = number of non-hydrogen atoms).

Cluster	Compound	Structure	MW (Da)	Confirmed purity	Matching mass	ΔMW (Da)	pIC_{50} primary assay	Inhibition ABPP assay (%)	cLog P	tPSA ($Å^2$)	HBD	HBA	LipE	LE
**Singleton**	**1**	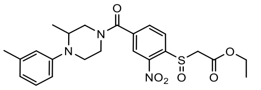	**474**	**Yes**	**Yes**	**-**	**6.2**	**86**	**3.09**	**135.8**	**0**	**7**	**3.1**	**0.30**
Benzoxazine derivative	**4**	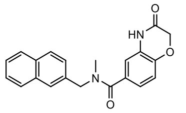	346	Yes	Yes	-	5.5	-3	3.16	58.6	1	3	2.3	0.29
Benzoxazine derivative	**5**	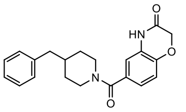	350	No	Yes	-	6.8	-2	2.92	58.6	1	3	3.8	0.36
Benzoxazine derivative	**6**	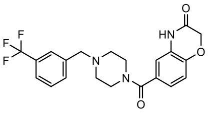	419	Yes	Yes	-	6.5	17	3.13	61.9	2	5	3.3	0.27
Benzoxazine derivative	**7**	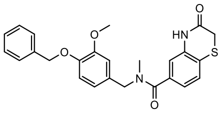	449	Yes	Yes	-	6.1	13	3.86	67.9	2	6	2.2	0.31
Benzoxazine derivative	**8**	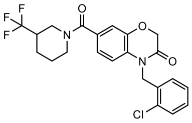	453	Yes	Yes	-	6.0	-6	3.56	49.9	1	3	2.4	0.29
Benzoxazine derivative	**9**	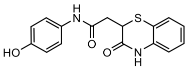	314	Yes	Yes	-	5.6	2	1.58	78.4	2	3	4.0	0.37
Carbamate	**10**	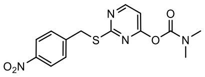	334	Yes	Yes	-	5.4	-4	2.12	106.1	1	3	3.3	0.30
Carbamate	**11**	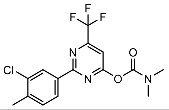	360	Yes	Yes	-	5.8	2	3.83	54.3	1	7	2.0	0.30
Fused imidazopyridine	**12**	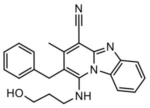	370	No	Yes	-	6.0	-13	5.16	71.7	1	4	0.9	0.27
Fused imidazopyridine	**13**	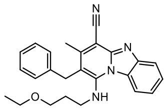	399	Yes	Yes	-	6.2	2	6.30	60.7	1	4	-0.1	0.27
Fused imidazopyridine	**14**	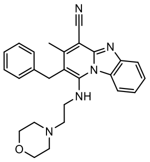	426	Yes	Yes	-	6.3	-11	5.77	63.9	1	4	0.6	0.33
Fused imidazopyridine	**15**	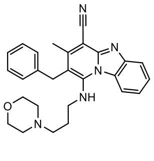	440	Yes	Yes	-	6.0	-12	6.04	63.9	2	4	0.0	0.29

38 **Supplementary Table 2 – Qualified hit list (continued).**

Cluster	Compound	Structure	MW (Da)	Confirmed purity	Confirmed mass	ΔMW (Da)	pIC_{50} primary assay	Inhibition ABPP assay (%)	cLog P	tPSA ($Å^2$)	HBD	HBA	LipE	LE
Imidazopiperidine	**16**	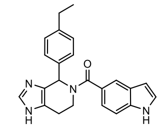	370	Yes	No†	+371	5.5	10	3.56	56.7	1	3	2.0	0.23
Imidazopiperidine	**17**	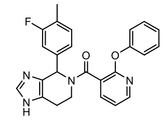	428	Yes	Yes	-	5.7	17	3.88	66.3	1	4	1.8	0.26
Imidazopiperidine	**18**	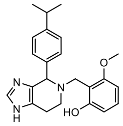	377	Yes	Yes	-	5.8	9	4.00	57.1	0	2	1.8	0.45
Imidazopiperidine	**19**	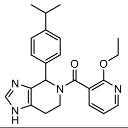	390	Yes	Yes	-	6.3	6	3.87	66.3	0	6	2.4	0.26
Imidazopyridines	**20**	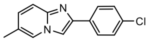	243	Yes	Yes	-	5.4	-10	4.72	15.6	0	4	0.7	0.33
Imidazopyridines	**21**	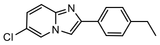	257	Yes	Yes	-	5.4	4	5.26	15.6	0	6	0.1	0.34
Imidazopyridines	**22**	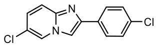	263	Yes	Yes	-	5.6	10	4.94	15.6	0	3	0.7	0.31
Naphtyl amide	**23**	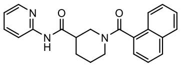	359	No	No	+113	5.7	-9	3.14	61.8	1	3	2.5	0.32
Naphtyl amide	**24**	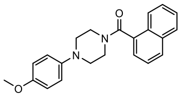	346	Yes	No	+28	5.8	51	3.96	32.8	0	2	1.8	0.35
Naphtyl amide	**25**	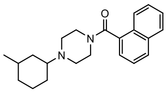	336	No	Yes	-	6.2	16	4.97	23.6	0	2	1.3	0.26
Naphtyl amide	**26**	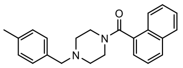	344	No	No	+45	4.9	-4	4.73	23.6	2	5	0.2	0.30
Naphtyl amide	**27**	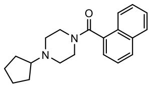	308	No	Yes	-	5.5	-11	3.77	23.6	0	2	1.7	0.35
Naphtyl amide	**28**	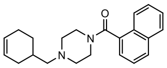	334	Yes	Yes	-	5.3	-7	4.55	23.6	2	4	0.7	0.29
Naphtyl amide	**29**	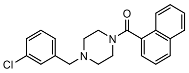	365	Yes	Yes	-	5.4	24	4.94	23.6	0	6	0.5	0.27
Naphtyl amide	**30**	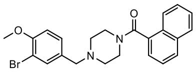	439	Yes	Yes	-	6.2	-17	4.92	32.8	4	6	1.3	0.26
Naphtyl amide	**31**	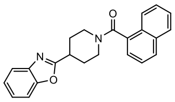	356	Yes	Yes	-	5.7	69	3.42	41.9	1	4	2.3	0.33

Supplementary Table 2 – Qualified hit list (continued).

Cluster	Compound	Structure	MW (Da)	Confirmed purity	Confirmed mass	ΔMW (Da)	pIC_{50} primary assay	Inhibition ABPP assay (%)	cLog P	tPSA ($Å^2$)	HBD	HBA	LipE	LE
Phenyl thiazole	**32**	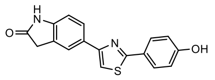	308	Yes	Yes	-	5.8	1	2.96	61.7	1	6	2.9	0.25
Phenyl thiazole	**33**	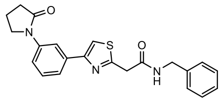	391	Yes	No	+15	5.9	11	3.12	61.8	2	5	2.8	0.29
Piperidine amide	**34**	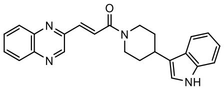	382	No	Yes	-	5.6	49	3.43	57.1	1	5	2.2	0.30
Piperidine amide	**35**	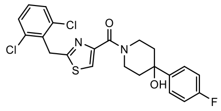	465	Yes	Yes	-	5.6	16	3.93	52.9	0	2	1.7	0.30
Piperidine amide	**36**	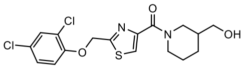	401	Yes	Yes	-	5.8	5	2.13	62.1	2	5	3.7	0.25
Singleton	**37**	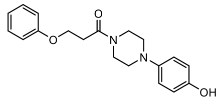	326	No	Yes	-	5.6	-1	2.97	53.0	0	9	2.6	0.28
Singleton	**38**	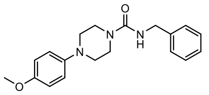	325	Yes	Yes	-	5.5	-6	3.39	44.8	0	4	2.1	0.80
Singleton	**39**	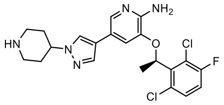	450	Yes	Yes	-	5.5	18	4.29	75.2	1	2	1.2	0.47
Singleton	**40**	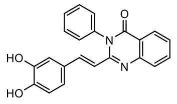	356	Yes	Yes	-	5.5	12	4.08	73.1	1	5	1.5	0.30
Singleton	**41**	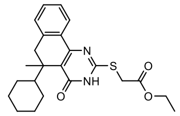	413	Yes	Yes	-	4.7	-3	5.59	67.8	3	3	-0.9	0.36
Singleton	**42**	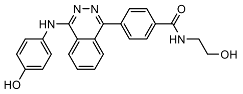	400	No	Yes	-	5.7	2	3.18	106.3	0	2	2.5	0.42
Singleton	**43**	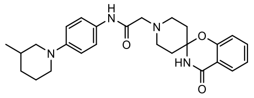	449	Yes	Yes	-	6.0	6	3.29	73.9	0	2	2.7	0.46
Singleton	**44**	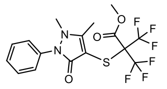	428	No	Yes	-	5.5	7	2.94	49.9	1	3	2.6	0.42
Singleton	**45**	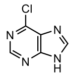	155	Yes	No	+205	5.7	0	-1.12	51.2	1	3	6.8	0.34

Supplementary Table 2 – Qualified hit list (continued).

Cluster	Compound	Structure	MW (Da)	Confirmed purity	Confirmed mass	ΔMW (Da)	pIC_{50} primary assay	Inhibition ABPP assay (%)	cLog P	tPSA ($Å^2$)	HBD	HBA	LipE	LE
Singleton	**46**	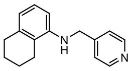	238	Yes	Yes	-	6.0	62	3.16	24.4	0	2	2.9	0.29
Singleton	**47**	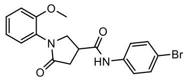	389	Yes	Yes	-	5.9	46	4.32	58.6	0	3	1.6	0.31
Sulfonamide	**48**	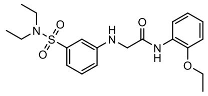	406	Yes	Yes	-	5.5	18	3.20	87.7	2	4	2.3	0.31
Sulfonamide	**49**	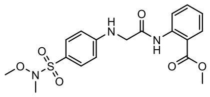	407	No	Yes	-	6.1	-7	1.94	114.0	1	6	4.2	0.28
Sulfonamide	**50**	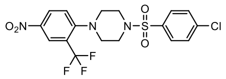	450	No	No	+22	6.3	13	5.08	92.4	1	6	1.2	0.26
Sulfonamide	**51**	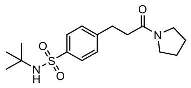	338	Yes	Yes	-	6.9	32	1.54	66.5	0	3	5.3	0.30
Sulfonamide	**52**	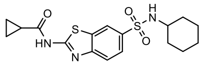	379	No	No	+328	5.5	0	4.20	87.6	2	7	1.3	0.26

Supplementary Table 3 – Data collection and refinement statistics for human MAGL LEI-515 complex.

	hMAGL-LEI-515 complex
**Data collection**	
Space group	$C222_1$
a, b, c (Å)	91.62, 127.57, 60.36
α, β, γ (°)	90, 90, 90
Resolution (Å)	1.55 (1.65-1.55)
R_{sym} or R_{merge}	0.063 (0.78)
$I / \sigma I$	11.69 (1.13)
Completeness (%)	99.6 (99.4)
$CC_{1/2}$	0.99/0.59
Redundancy	6.62 (6.16)
**Refinement**	
Resolution (Å)	48.3 – 1.55
No. reflections	48898
R_{work} / R_{free}	15.43/18.03
**No. atoms**	
Protein	2299
Water	325
Ligand	34
**B-factors (Å^2)**	
Protein	25.56
Water	47.53
Ligand	33.07
**R.m.s. deviations**	
Bond lengths (Å)	0.016
Bond angles (°)	1.999
PDB code	8AQF

*Values in parentheses are for highest resolution shell

Supplementary Table 4 – Off-target pharmacology of LEI-515

Targets	LEI-515 (µM)	% Inhibition of Control Specific Binding		
		1st	2nd	Mean
A2A(h) (agonist radioligand)`	10	8.1	15.2	11.7
α1A(h) (antagonist radioligand)	10	7.3	-0.3	3.5
α2A(h) (antagonist radioligand)	10	8.3	28.3	18.3
β1(h) (agonist radioligand)	10	0.9	12.3	6.6
β2(h) (antagonist radioligand)	10	25.6	28.0	26.8
BZD (central) (agonist radioligand)	10	-32.5	-13.0	-22.7
CB1(h) (agonist radioligand)	10	18.7	25.8	22.3
CB2(h) (agonist radioligand)	10	-8.9	-14.9	-11.9
CCK1 (CCKA) (h) (agonist radioligand)	10	13.3	25.8	19.5
D1(h) (antagonist radioligand)	10	2.1	10.2	6.2
D2S(h) (agonist radioligand)	10	-3.4	7.7	2.2
ETA(h) (agonist radioligand)	10	-5.9	-10.1	-8.0
NMDA (antagonist radioligand)	10	11.1	1.6	6.3
H1(h) (antagonist radioligand)	10	12.5	3.7	8.1
H2(h) (antagonist radioligand)	10	-26.7	-25.3	-26.0
MAO-A (antagonist radioligand)	10	4.9	7.4	6.1
M1(h) (antagonist radioligand)	10	-25.4	-33.6	-29.5
M2 (h) (antagonist radioligand)	10	-6.8	-1.7	-4.2
M3(h) (antagonist radioligand)	10	-24.6	-15.2	-19.9
N neuronal α4β2 (h) (agonist radioligand)	10	-7.8	-13.2	-10.5
δ (DOP) (h) (agonist radioligand)	10	53.0	43.7	48.3
kappa (h) (KOP) (agonist radioligand)	10	62.3	56.8	59.5
kappa (h) (KOP) (agonist radioligand)	1	12.8	30.6	21.7
µ (MOP) (h) (agonist radioligand)	10	25.6	15.2	20.4
5-HT1A(h) (agonist radioligand)	10	8.8	12.4	10.6
5-HT1B (h) (antagonist radioligand)	10	22.7	25.6	24.2
5-HT2A(h) (agonist radioligand)	10	-5.5	-5.5	-5.5
5-HT2B(h) (agonist radioligand)	10	30.7	24.4	27.6
5-HT3(h) (antagonist radioligand)	10	-8.5	-7.8	-8.1
GR (h) (agonist radioligand)	10	29.5	30.8	30.2
AR(h) (agonist radioligand)	10	-1.1	-13.0	-7.0
V1a(h) (agonist radioligand)	10	13.1	6.8	9.9
Ca2+ channel (L, dihydropyridine site) (antagonist radioligand)	10	58.2	50.6	54.4
Ca2+ channel (L, dihydropyridine site) (antagonist radioligand)	1	18.1	19.6	18.9
Potassium Channel hERG (human)- $[^3H]$ Dofetilide	10	8.5	3.0	5.8
KV channel (antagonist radioligand)	10	-1.2	-8.1	-4.7

Na+ channel (site 2) (antagonist radioligand)	10	61.6	49.5	55.6
Na+ channel (site 2) (antagonist radioligand)	1	12.6	-1.9	5.3
norepinephrine transporter(h) (antagonist radioligand)	10	24.9	25.7	25.3
dopamine transporter(h) (antagonist radioligand)	10	47.6	42.8	45.2
5-HT transporter (h) (antagonist radioligand)	10	10.3	-2.0	4.2
COX1(h)	10	13.4	-2.5	5.4
COX2(h)	10	20.3	-1.4	9.5
PDE3A (h)	10	-20.5	-33.8	-27.2
PDE4D2 (h)	10	-12.4	-20.9	-16.6
Lck kinase (h)	10	-9.2	-1.4	-5.3
acetylcholinesterase (h)	10	4.4	5.4	4.9

Supplementary Table 5 – PK parameters of LEI-515 in mice after p.o. and i.v. administration. CL = clearance. V_{ss} = volume of distribution at steady state. $t_{1/2}$ = half-life. C_{max} = maximum plasma drug concentration. t_{max} = time to reach C_{max}. AUC = area under plasma concentration time curve. F_{po} = bioavailability.

	p.o.	i.v.
Dose (mg/kg)	10	10
CL (ml/min/kg)	-	35
V_{ss} (L/kg)	-	2.1
$t_{1/2}$ (h)	-	4.5
C_{max} (nM)	2433	-
t_{max} (h)	0.5	
AUC (h*nmol/L)	7330	-
F_{po} (%)	81	
C_{brain}/C_{plamsa}	0.01	

Supplementary Table 6 – LC-MS Standards and deuterium labeled internal standards for lipidomics analysis. Q1 and Q3 are optimized precursor ion and product ion respectively and expressed as m/z. DP and CE are declustering potential (volt) and collision energy (Volt) respectively.

Abbreviation	Name	Q1	Q3	DP, CE	Polarity
**1 & 2-AG (20:4)**	1-Arachidonoyl Glycerol	379.21	287.2	45, 10	+
**1-LG (18:2)**	1-Linoleoyl Glycerol	355.34	246	48, 10	
**2-LG (18:2)**	2-Linoleoyl Glycerol	357.34	247.5	48, 10	
**2-OG (18:1)**	2-Oleoyl Glycerol	357.34	247.5	40, 12	
**1-OG (18:1)**	1-Oleoyl Glycerol	357.34	247.5	40, 12	
**AEA (20:4)**	Arachidonoyl Ethanolamide	348.40	62.02	35, 16	
**DEA (22:4)**	Docosatetraenoyl Ethanolamide	376.38	61.92	55, 18	
**DGLEA (18:3)**	Dihomo-γ-Linolenoyl Ethanolamide	350.38	61.98	40, 14	
**DHEA (22:6)**	Docosahexaenoyl Ethanolamide	372.38	62.01	50, 14	

**EPEA (20:5)**	Eicosapentaenoyl Ethanolamide	346.34	61.98	36, 16	
**LEA (18:2)**	Linoleoyl Ethanolamide	324.34	61.98	35, 14	
**OEA (18:1)**	Oleoyl Ethanolamide	326.4	62.01	45, 16	
**PDEA (15:0)**	Pentadecanoyl Ethanolamide	286.34	62.01	45, 12	
**PEA (16:0)**	Palmitoyl Ethanolamide	300.34	61.98	42, 14	
**POEA (16:1)**	Palmitoleoyl Ethanolamide	298.34	62.01	45, 14	
**SEA (18:0)**	Stearoyl Ethanolamide	328.38	61.98	45, 16	
**AA (20:4)**	Arachidonic Acid	302.28	259.3	-40, -12	-
**PA (FA 16:0)**	Palmitic Acid	255.33	237.24	-50, -20	
**SA (FA 18:0)**	Stearic Acid	283.34	265.31	-60, -22	
**OA (FA 18:1)**	Oleic Acid	281.34	263.31	-50, -20	
**LA (FA 18:2)**	Linoleic Acid	279.34	261.25	-64, -16	
**GLA (FA 18:3)**	γ-Linolenic Acid	277.3	58	-60, -20	
**ETA (FA 20:3. (ω−3)**	11(Z).14(Z).17(Z)-Eicosatrienoic Acid	305.28	306.09	-60, -18	
**DGLA (FA 20:3. (ω−6)**	Dihomo-γ-Linolenic Acid (20:3)	305.28	306.03	-66, -18	
**EPA (FA 20:5.(ω−3)**	Eicosapentaenoic Acid	301.34	257.3	-60, -10	
**DHA (FA 22:6. (ω−3)**	Docosahexaenoic Acid	327.28	283.31	-60, -10	
**2-AG-d8 (20:4)**	2-Arachidonoyl Glycerol-d8	387.38	294.2	45, 10	+
**AEA-d8 (20:4)**	Arachidonoyl Ethanolamide-d8	356.38	62.79	35, 16	
**DHEA-d4 (22:6)**	Docosahexaenoyl Ethanolamide-d4	376.38	66.01	50, 14	
**LEA-d4 (18:2)**	Linoleoyl Ethanolamide-d4	328.34	66.01	35, 16	
**OEA-d4 (18:1)**	Oleoyl Ethanolamide-d4	330.38	66.01	45, 16	
**PEA-d5 (16:0)**	Palmitoyl Ethanolamide-d5	305.34	61.98	42, 16	
**SEA-d3 (18:0)**	Stearoyl Ethanolamide-d3	331.38	61.91	45, 16	
**EPEA-d4 (20:5)**	Eicosapentaenoyl Ethanolamide-d4	350.34	66.08	36, 18	
**AA-d8 (20:4)**	Arachidonic Acid-d8	311.34	267.30	-40, -12	-
**PA (16:0)-d31**	Palmitic Acid-d31	286.5	266.37	-40, -22	

Supplementary Table 7 – LC-MS Standards and deuterium labeled internal standards for *in vivo* lipidomics analysis.

Analyte	MS-Mode	Mass transition	Cone voltage (V)	Collision energy (eV)
Arachidonic acid (AA) C20:4	ES-	303.14 > 259.16	20	16
Arachidonic acid (AA) C20:4-d8	ES-	311.19 > 267.10	10	15
2-Arachidonylglycerol (2-AG)	ES+	379.24 > 287.21	40	14
2-Arachidonylglycerol (2-AG-d5)	ES+	384.24 > 287.21	40	14

Supplementary Table 8 – Summary of Three-way ANOVA results

Figure	Source of Variation
**6b**	Three-way ANOVA revealed significant main effects of drug treatment (p<0.0001) and time (p<0.0001) and the interaction between treatment and time was significant (p<0.0001). No main effect of sex (p=0.1814) or interaction with sex (Time x Sex: p=0.3212; Sex x Treatment: p=0.873; Time x Sex x Treatment: p=0.7577) was detected. See also Fig. S7a.
**6c**	Three-way ANOVA revealed main effects of drug treatment (p<0.0001) and time (p=0.0357) but no main effect of sex (p=0.3512) or interaction with sex or time (Sex x Treatment: p=0.1288; Time x Sex: p=0.4493; Time x Treatment 0.0743; Time x Sex x Treatment: p=0.6927) was detected throughout the period of chronic (p.o.) dosing. Efficacy was preserved at least 4 days following termination of oral dosing; three-way ANOVA revealed main effects of drug treatment (p<0.0001), time (p=0.0037) and their interaction (p=0.0007) but no main effect of sex (Sex: p=0.4215) or interaction with sex (Sex x Treatment: p=0.6342; Time x Sex: p=0.6380; Time x Sex x Treatment: p=0.9346) was detected across the 10-day observation interval following termination of chronic dosing. See also Fig. S7c.
**6e**	Three-way ANOVA revealed significant main effects of drug treatment (p<0.0001) and time (p<0.0001) and their interaction (p<0.0001; see also Fig. S7a) but no main effect of sex (p=0.1269) or interaction with sex (Sex x Treatment: p= 0.1547; Time x Sex: p=0.6117; Time x Sex x Treatment: p=0.5945) was detected. ****p<0.0001 LEI-515 vs. vehicle control groups. See also Fig. S7b.
**6f**	Three-way ANOVA revealed a main effect of drug treatment (p<0.0001) and an interaction between drug treatment, time and sex (p=0.049), but no other main effects (Time: p=0.6512; Sex: p=0.206) or interactions (Time x Sex: p=0.2165; Time x Treatment: p=0.8939; Sex x Treatment: p=0.3299) were detected throughout the period of chronic (i.p.) dosing. Efficacy was preserved at least 4 days following termination of i.p. dosing; Three-way ANOVA revealed significant main effect of drug treatment (p<0.0001) and time (p<0.0001) and the interaction between drug treatment and time was significant (p<0.0001) but no main effect of sex (p=0.5045), or interaction with sex (Sex x Time: p=0.1657; Sex x Treatment p=0.4588; Time x Sex x Treatment p=0.5596) was detected. See also Fig. S7d.

Supplementary Table 9: Summary of P values obtained from Two-way ANOVA justifying pooling of sexes in Supplementary Figure S7.

Figure	Groups	Time	Treatment	Interaction
**6B (p.o.)**	VEH (M) vs. VEH (F)	0.635	0.2021	0.1676
**6B (p.o.)**	LEI-515 (M) vs. LEI-515 (F)	p<0.0001	0.4809	0.7937
**6B (p.o.)**	VEH (M+F) vs. LEI-515 (M+F)	p<0.0001	p<0.0001	p<0.0001
**6C Day 1-10 (p.o.)**	VEH (M) vs. VEH (F)	0.5209	0.6045	0.9604

**6C Day 1-10 (p.o.)**	LEI-515 (M) vs. LEI-515 (F)	0.0404	0.1516	0.4607
**6C Day 1-10 (p.o.)**	VEH (M+F) vs. LEI-515 (M+F)	0.0371	P<0.0001	0.0803
**6C Day 11-20 (p.o.)**	VEH (M) vs. VEH (F)	0.9385	0.2896	0.8983
**6C Day 11-20 (p.o.)**	LEI-515 (M) vs. LEI-515 (F)	0.0003	0.8415	0.6916
**6C Day 11-20 (p.o.)**	VEH (M+F) vs. LEI-515 (M+F)	0.0024	p<0.0001	0.0004
**6E (i.p.)**	VEH (M) vs. VEH (F)	0.6683	0.9325	0.972
**6E (i.p.)**	LEI-515 (M) vs. LEI-515 (F)	p<0.0001	0.0647	0.4224
**6E (i.p.)**	VEH (M+F) vs. LEI-515 (M+F)	p<0.0001	p<0.0001	p<0.0001
**6F Day 1-10 (i.p.)**	VEH (M) vs. VEH (F)	0.8236	0.7543	0.1814
**6F Day 1-10 (i.p.)**	LEI-515 (M) vs. LEI-515 (F)	0.6749	0.2036	0.0706
**6F Day 1-10 (i.p.)**	VEH (M+F) vs. LEI-515 (M+F)	0.7086	p<0.0001	0.8744
**6F Day 11-20 (i.p.)**	VEH (M) vs. VEH (F)	0.3435	0.9494	0.5984
**6F Day 11-20 (i.p.)**	LEI-515 (M) vs. LEI-515 (F)	p<0.0001	0.3865	0.1964
**6F Day 11-20 (i.p.)**	VEH (M+F) vs. LEI-515 (M+F)	p<0.0001	p<0.0001	p<0.0001

Abbreviations: M, male; F, female; p.o., oral; i.p., intraperitoneal
Source data and all statistical analyses are provided as a Source data file.

Figure S1

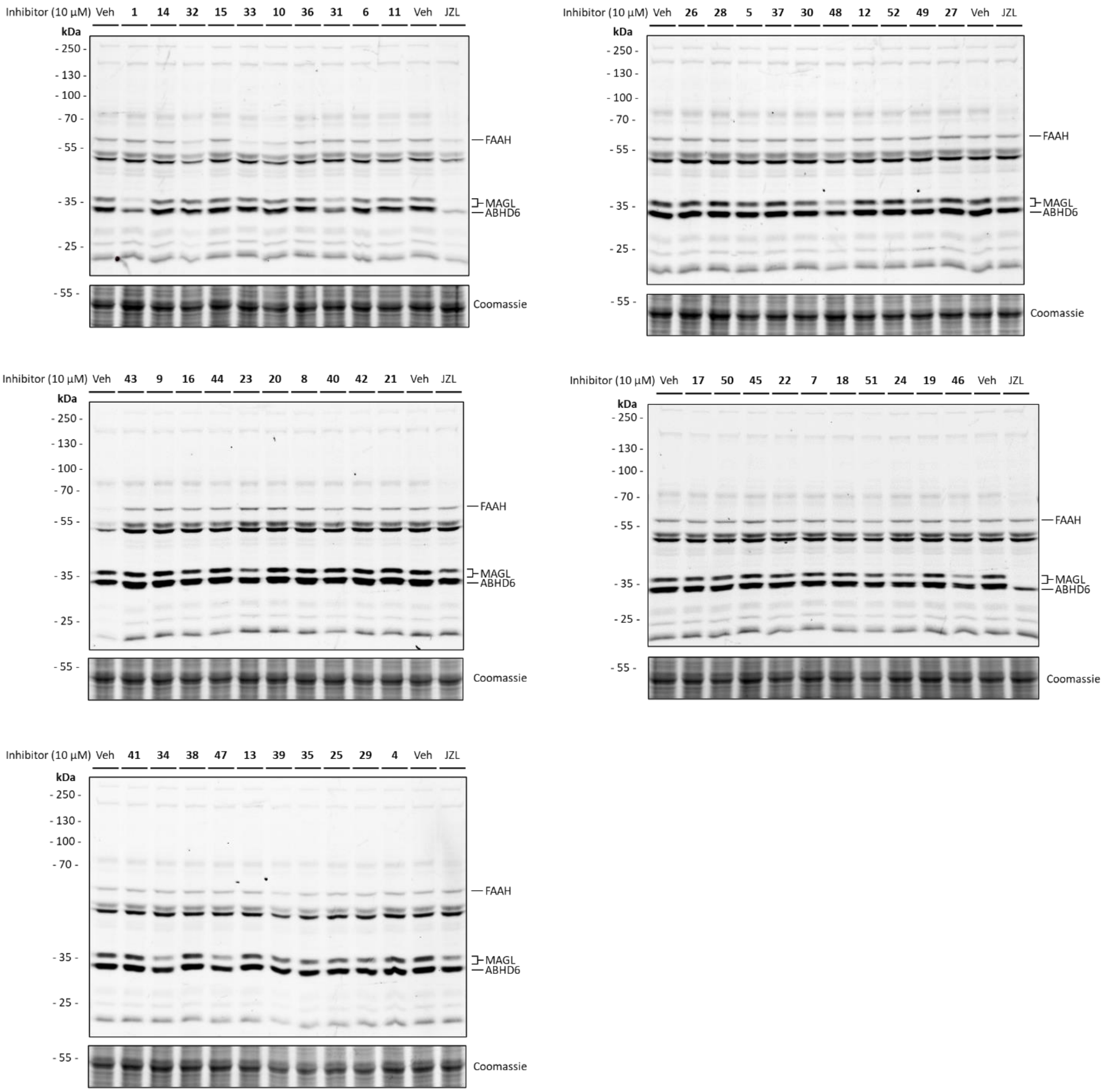

Supplementary Figure 1 – Orthogonal competitive ABPP assay for hit validation and selectivity assessment. ABPP was performed on mouse brain membrane proteome using FP-TAMRA. Proteome was pre-incubated with inhibitor (10 µM, 30 min), followed by incubation with FP-TAMRA (100 nM, 10 min). JZL184 was included as positive control on all gels. The data shown is n=1 due to limited amount of sample of the hits of which some was used for LC-MS analysis confirming the compound identity.

Figure S2

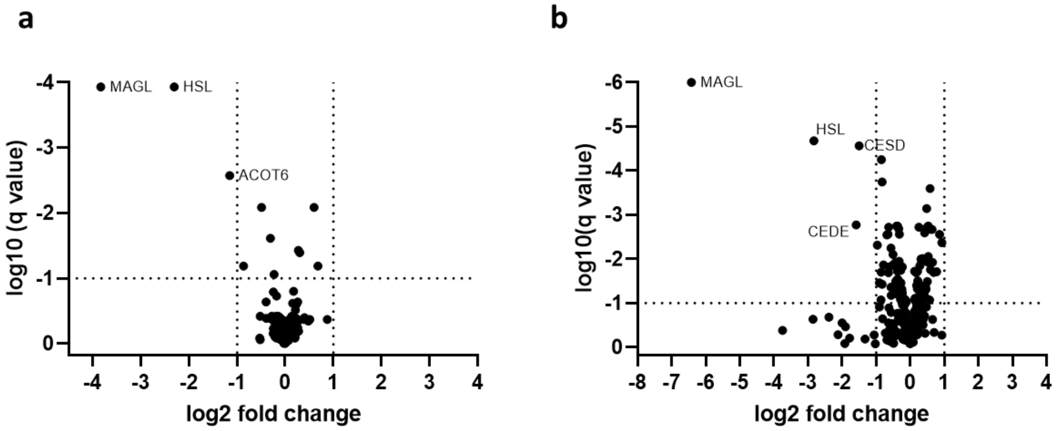

Supplementary Figure 2 – Chemical proteomics-based selectivity profiles of LEI-515. LEI-515 (1 µM, 30 min, 37°C) was incubated with mouse brain (**a**) and liver (**b**) proteomes using broad-spectrum probes MB108 and FP-biotin (10 µM, 60 min, 37°C) (n = 4). Data was analysed in GraphPad Prism 9.0 (multiple t-test).

Figure S3

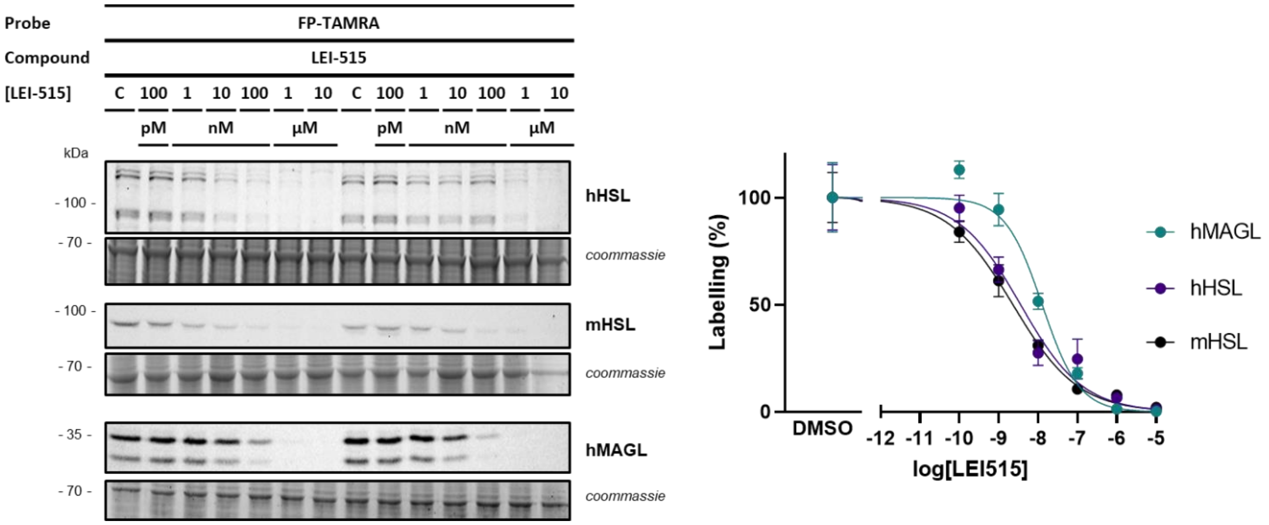

Supplementary Figure 3 – ABPP potency assessment of LEI-515 for human MAGL, human HSL and mouse HSL. Dose-response treatment of LEI-515 on hMAGL (pIC_{50} = 7.90 ± 0.11), hHSL (pIC_{50} = 8.44 ± 0.15), and mHSL (pIC_{50} = 8.64 ± 0.11), overexpression lysate using FP-TAMRA (10 min, 100 nM), C = DMSO control. Fluorescence and Coomassie intensities were quantified using ImageLab 6.0 software (Bio-Rad) and normalized log-transformed data was fitted using the variable slope dose-response function in GraphPad Prism 9.0 (n=4 biologically independent experiments). Data are presented as mean ± SEM.

Figure S4

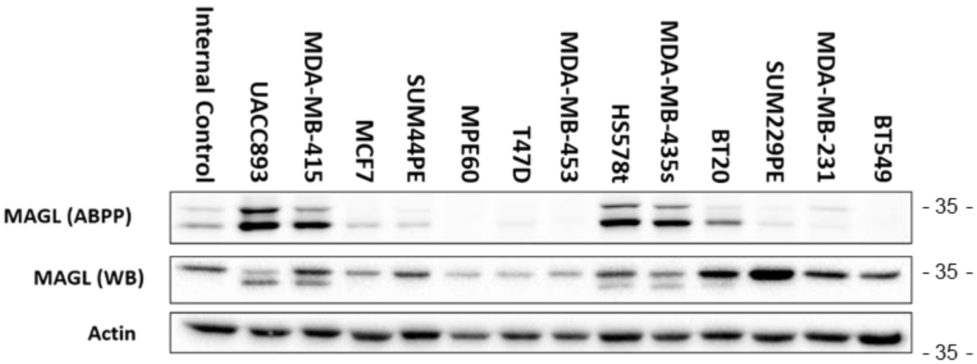

Supplementary Figure 4 – Profiling of MAGL activity and protein level in a panel of 13 breast cancer cell lines. ABPP was performed on cell lysates (2mg/mL) using LEI-463-Cy5 (100 nM, 15 min). Actin was used as a loading control. MAGL antibody: ab24701 (1:200 dilution). n=3.

Figure S5

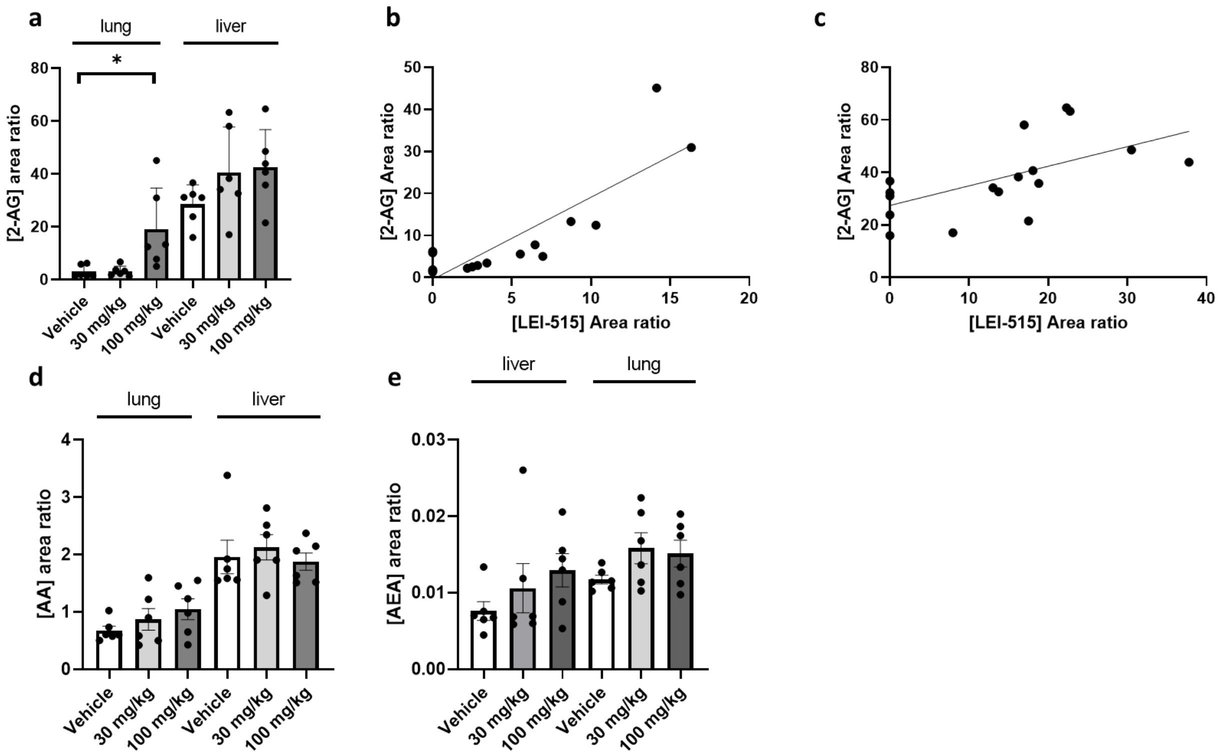

Supplementary Figure 5 – *In vivo* 2-AG levels in lung and liver after LEI-515 treatment. (**a**) 2-AG levels in C57BL/6J mice lung and liver (100 mg/kg p = 0.0158) after oral administration (30 or 100 mg/kg) of LEI-515. (n = 4 for vehicle and n = 6 for LEI-515). High variability in 2-AG levels was observed in these tissues. Therefore, 2-AG levels in (**b**) lung ($R^2 = 0.73$, $p < 0.001$) and (**c**) liver ($R^2 = 0.36$, $p = 0.0084$) were plotted against quantified LEI-515 levels. An increase in 2-AG levels was associated with increasing LEI-515 levels. (**d-e**) AA and AEA levels in lung and liver tissue (n = 4 for vehicle and n = 6 for LEI-515).. Statistical analysis: One-way ANOVA and Dunnett post-hoc test (**a, d** and **e**) or linear regression (**b** and **c)**. *p<0.05 vs. vehicle control. Data are presented as mean ± SEM.

Figure S6

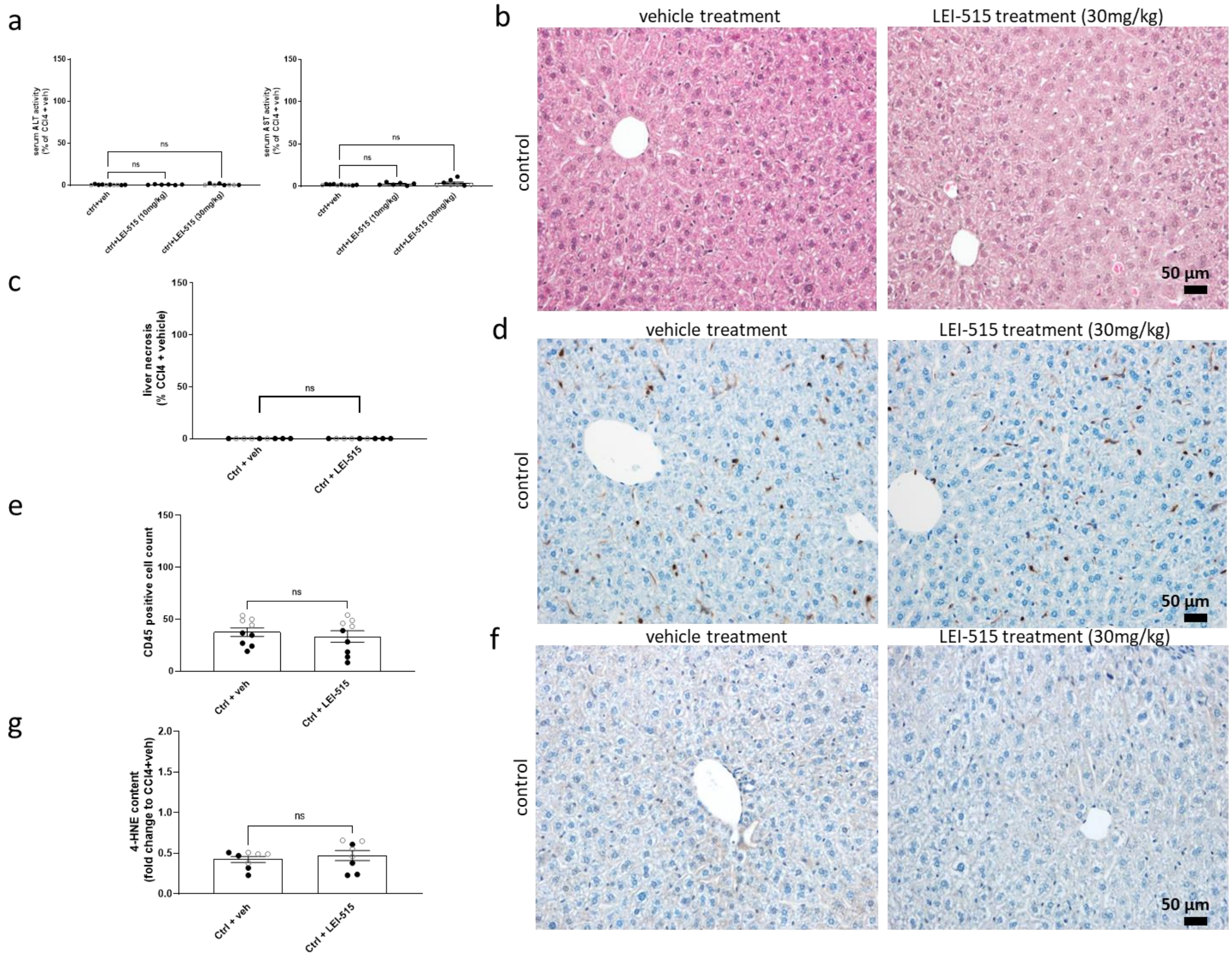

Supplementary Figure 6 – LEI-515 has no effects on liver function in control animals. (**a**) Effect of vehicle treatment (n=10) or LEI-515 (10 and 30 mg/kg i.p., n=6 or 8/group, respectively) on liver transaminases [alanine aminotransferase (ALT) and aspartate aminotransferase (AST) respectively], (**b-c**) histopathological liver necrosis in control groups and its quantification (n=9/group), (**d-e**) CD45+ leukocyte infiltration in control groups and its quantification (n=9/group), (**f-g**) lipid peroxidation (4-HNE) in control groups and its quantification (n = 8/group). Data are presented as mean ± SEM. Open symbols represent female, whereas closed symbols show male subjects. Statistical analysis: One-way ANOVA and Dunnett post-hoc test (**a**) or unpaired two-tailed t-test (**c**; **e**; **g**).

Figure S7

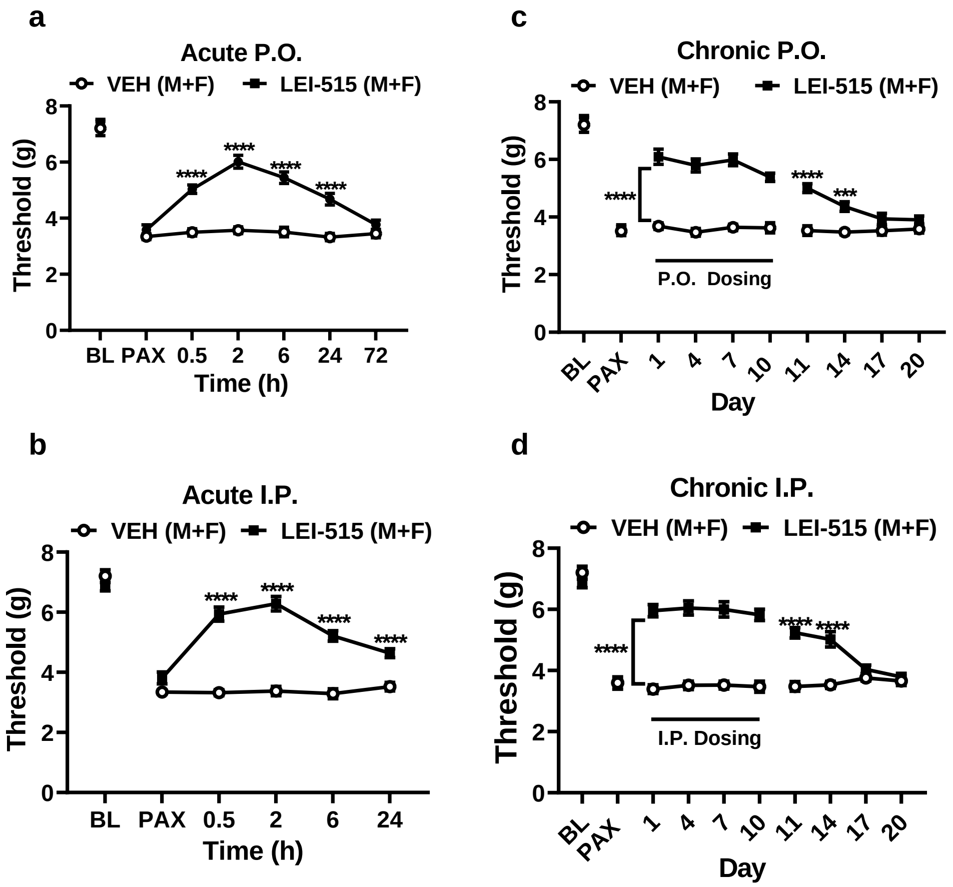

Supplementary Figure 7 – The peripherally restricted MAGL inhibitor LEI-515, administered p.o. or i.p., suppresses paclitaxel-induced mechanical hypersensitivity in mixed sex groups. Acute LEI-515 (10 mg/kg) suppressed paclitaxel-induced mechanical sensitivity following (**a**) oral or (**b**) i.p. administration. Efficacy was observed from 0.5-24 h for each route of administration. (**a-b**) ANOVA revealed main effects of treatment ($p<0.0001$) and time ($p<0.0001$) and the interaction ($p<0.0001$) was significant. ****$p<0.0001$ vs. vehicle (Two-way ANOVA, Sidak's multiple comparison test). (**c**) Once daily dosing with LEI-515 (10 mg/kg/day p.o. for 10 days) suppressed paclitaxel-induced mechanical hypersensitivity without loss of efficacy during the period of oral drug delivery; Two-way ANOVA revealed a main effect of treatment ($p<0.0001$) and time ($p=0.0371$) and the interaction ($p=0.0803$) was not significant. Efficacy was preserved at least 4 days following termination of p.o. dosing; Two-way ANOVA revealed a main effect of drug treatment ($p<0.0001$), time ($p=0.0024$) and the interaction ($p=0.0004$) was significant across the ten-day observation interval following termination of drug delivery. (**d**) Once daily dosing with LEI-515 (10 mg/kg/day i.p. for 10 days) suppressed paclitaxel-induced mechanical hypersensitivity without loss of efficacy during the period of drug delivery. Two-way ANOVA revealed a main effect of drug treatment ($p<0.0001$), but no main effect of time ($p=0.7086$) or interaction ($p=0.8744$) was detected across the period of i.p. drug delivery. Efficacy was preserved at least 4 days following termination of i.p. dosing; Two-way ANOVA revealed a main effect of treatment ($p<0.0001$) and time ($p<0.0001$) and the interaction ($p<0.0001$) was significant. (**a-d**) ****$p<0.0001$, ***$p<0.001$ vs. vehicle control. See Supplementary Table 9 for statistical results comparing sexes that were pooled for each treatment. (**a, c**) n=16 mixed sex vehicle (n=8 per sex), n=15 mixed sex LEI-515 (n=7-8 per sex). (**b, d**) n=15 mixed sex vehicle (n=7-8 per sex), n=16 mixed sex LEI-515 (n=8 per sex). (**a-d**) All data are presented as mean ± SEM. Statistical analysis: Two-way ANOVA with Tukey and Sidak Multiple Comparison tests. Source data are provided as Source data file.

Synthetic procedures

General remarks

All reactions were performed using oven or flame-dried glassware and dry solvents. Reagents were purchased from Sigma Aldrich, Acros and Merck and used without further purification unless noted otherwise. All moisture sensitive reactions were performed under an argon or nitrogen atmosphere. Traces of water were removed from starting compounds by co-evaporation with toluene. Reactions were followed by thin layer chromatography and was performed using TLC Silica gel 60 F_{245} on aluminums sheets. Compounds were visualized using a $KMnO_4$ stain (K_2CO_3 (40 g), $KMnO_4$ (6 g), H_2O (600 mL) and 10% NaOH (5 mL)). ^{1}H- and ^{13}C-NMR spectra were recorded on a Bruker AV-400, 500, 600 or 850 using $CDCl_3$ or CD_3OD as solvent, unless stated otherwise. Chemical shift values are reported in ppm with tetramethylsilane or solvent resonance as the internal standard ($CDCl_3$: δ 7.26 for ^{1}H, δ 77.16 for ^{13}C, CD_3OD: δ 3.31 for ^{1}H, δ 49.00 for ^{13}C). Data are reported as follows: chemical shifts (δ), multiplicity (s = singlet, d = doublet, dd = double doublet, td = triple doublet, t = triplet, q = quartet, quinted = quint, br = broad, m = multiplet), coupling constants *J* (Hz), and integration. LC-MS measurements were performed on a Thermo Finnigan LCQ Advantage Max ion-trap mass spectrometer (ESI+) coupled to a Surveyor HPLC system (Thermo Finnigan) equipped with a standard C18 (Gemini, 4.6 mmD × 50 mmL, 5 µm particle size, Phenomenex) analytical column and buffers A: H_2O, B: ACN, C: 0.1% aq. TFA. Preparative HPLC purification was performed on a Waters Acquity Ultra Performance LC with a C18 column (Gemini, 150 × 21.2 mm, Phenomenex). Diode detection was done between 210 and 600 nm. Gradient: ACN in (H_2O + 0.2% TFA). High-resolution mass spectra (HRMS) were recorded on a Thermo Scientific LTQ Orbitrap XL.

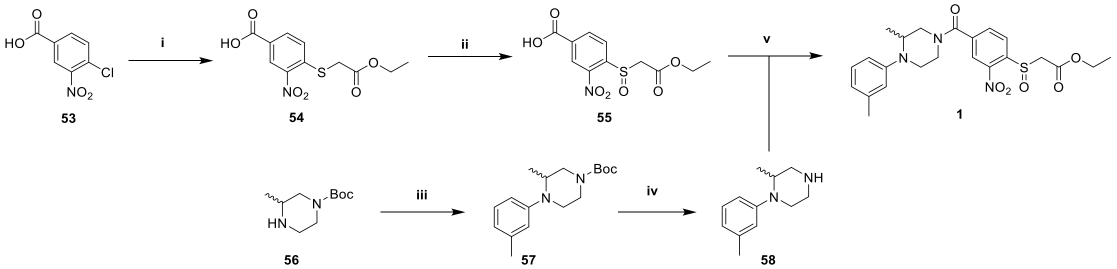

Scheme 1. Synthesis route for hit **1**. Reagents and conditions: i) ethyl 2-mercaptoacetate, pyridine, 115 °C. ii) Oxone, MeOH / H_2O. iii) sodium *tert*-butoxide, BINAP, $Pd(OAc)_2$, 1,4-dioxane, 85 °C. iv) TFA, DCM. v) HATU, DiPEA, DCM.

4-((2-Ethoxy-2-oxoethyl)thio)-3-nitrobenzoic acid (**54**)

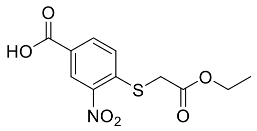

To a solution of 4-chloro-3-nitrobenzoic acid (1.26 mg, 6.24 mmol, 1.50 eq.) in pyridine (5 mL) was added ethyl mercaptoacetate (0.5 g, 4.16 mmol, 1.00 eq.) and the mixture was heated to 115 °C overnight in an oil bath. The reaction progress was monitored by TLC analysis. Upon full conversion of the starting materials, the mixture was allowed to cool to rt and the pH was adjusted to 1 with 1M HCl solution. The precipitate was filtered and the solid were washed with water to provide the product (1.01 g, 3.54 mmol, 85%). ^{1}H NMR (400 MHz, Methanol-*d*4) δ 8.73 (d, *J* = 1.9 Hz, 1H), 8.16 (dd, *J* = 8.5, 1.9 Hz, 1H), 7.66 (d, *J* = 8.5 Hz, 1H), 4.20 (q, *J* = 7.1 Hz, 2H), 4.00 (s, 2H), 1.25 (t, *J* = 7.1 Hz, 3H). ^{13}C NMR (101 MHz, Methanol-*d*4) δ 170.38, 167.34, 143.24, 135.02, 129.44, 128.46, 128.08, 63.20, 35.65, 14.53.

4-((2-Ethoxy-2-oxoethyl)sulfinyl)-3-nitrobenzoic acid (**55**)

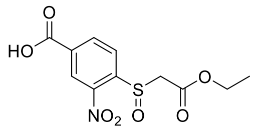

To a cooled solution of 4-((2-ethoxy-2-oxoethyl)thio)-3-nitrobenzoic acid (285 mg, 1.00 mmol, 1.00 eq.) in methanol (13 mL) was dropwise added a solution of Oxone (62 mg, 1.00 mmol, 1.00 eq.) in water (4 mL) and the reaction mixture was stirred at rt for 2.5 h. The reaction progress was monitored by TLC analysis. Upon full conversion of the starting materials, the mixture was diluted with water and extracted with DCM. The combined organic layers were washed with brine, dried ($MgSO_4$), filtered and concentrated under reduced pressure. The residue was purified by silica gel column chromatography (MeOH/DCM, 1% to 2%) to afford the product (210 mg, 0.70 mmol, 70%). ^{1}H NMR (400 MHz, Methanol-*d*4) δ 8.88 (d, *J* = 1.6 Hz, 1H), 8.61 (dd, *J* = 8.2, 1.6 Hz, 1H), 8.34 (d, *J* = 8.2 Hz, 1H), 4.36 (d, *J* = 14.4 Hz, 1H), 4.29 – 4.13 (m, 2H), 3.82 (d, *J* = 14.5 Hz, 1H), 1.26 (t, *J* = 7.1 Hz, 3H). ^{13}C NMR (101 MHz, Methanol-*d*4) δ 166.82, 166.60, 147.30, 136.89, 136.55, 128.43, 127.26, 63.37, 61.32, 14.54.

tert-Butyl (R)-3-methyl-4-(m-tolyl)piperazine-1-carboxylate ((R)-57)

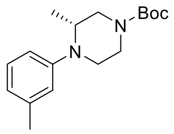

A mixture of 1-bromo-3-methylbenzene (1 g, 5.85 mmol, 1 eq.), *tert*-butyl (*R*)-3-methylpiperazine-1-carboxylate (1.17 g, 5.85 mmol, 1 eq.), Cs_2CO_3 (2.86 g, 8.77 mmol, 1.5 eq.), *rac*-BINAP (233 mg, 0.35 mmol, 0.06 eq.) and palladium diacetate (52.53 mg, 0.23 mmol, 0.04 eq.) in degassed 1,4-dioxane was heated to 85 °C under nitrogen atmosphere overnight. The reaction progress was monitored by TLC analysis. Upon full conversion of the starting materials, the mixture was diluted with DCM, washed with water, dried ($MgSO_4$), filtered and concentrated under reduced pressure. The residue was purified by silica gel column chromatography (pentane/diethyl ether,10/1) to give the product (1.05 g, 3.62 mmol, 62%).

tert-Butyl (S)-3-methyl-4-(m-tolyl)piperazine-1-carboxylate ((S)-57)

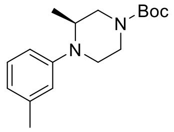

A mixture of 1-bromo-3-methylbenzene (100 mg, 0.59 mmol, 1 eq.), *tert*-butyl (*S*)-3-methylpiperazine-1-carboxylate (176 mg, 0.88 mmol, 1.5 eq.), Cs_2CO_3 (286 mg, 0.88 mmol, 1.5 eq.), rac-BINAP (23.25 mg, 0.04 mmol, 0.06 eq.) and palladium diacetate (5.25 mg, 0.02 mmol, 0.04 eq.) in degassed 1,4-dioxane was heated to 85 °C under nitrogen atmosphere overnight. The reaction progress was monitored by TLC analysis. Upon full conversion of the starting materials, the mixture was diluted with DCM, washed with water, dried ($MgSO_4$), filtered and concentrated under reduced pressure. The residue was purified by silica gel column chromatography (pentane/diethyl ether,10/1) to give the product (130 mg, 0.45 mmol, 77%). ^{1}H NMR (400 MHz, $CDCl_3$) δ 7.14 (t, *J* = 7.7 Hz, 1H), 6.71 – 6.67 (m, 3H), 4.04 – 3.66 (m, 3H), 3.44 – 2.99 (m, 4H), 2.30 (s, 3H), 1.48 (s, 9H), 0.98 (d, *J* = 6.5 Hz, 3H). ^{13}C NMR (101 MHz, $CDCl_3$) δ 155.13, 150.22, 138.86, 129.07, 120.93, 118.05, 114.33, 79.70, 51.65, 49.45, 48.25, 44.30, 28.50, 21.85, 12.25.

(*R*)-2-methyl-1-(*m*-tolyl)piperazine ((*R*)-58)

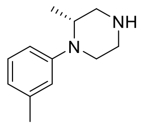

To a solution of **(*R*)-57** (0.75 g, 2.58 mmol, 1.00 eq.) in DCM (8 mL) was added 2 mL TFA and the mixture was stirred for 2 h at room temperature. The reaction progress was monitored by TLC analysis. Upon full conversion of the starting materials, the reaction mixture was diluted with DCM (20 mL), washed with saturated $NaHCO_3$ solution, dried ($MgSO_4$), filtered and concentrated under reduced pressure. The residue was purified by silica gel column chromatography (DCM/MeOH,10/1) to give the product (405 mg, 2.13 mmol, 82%). ^{1}H NMR (400 MHz, $CDCl_3$) δ 7.20 – 7.08 (m, 1H), 6.80 – 6.68 (m, 3H), 3.72 (m, 1H), 3.67 – 3.50 (br, 1H), 3.19 – 2.82 (m, 6H), 2.31 (s, 3H), 1.03 (d, *J* = 6.5 Hz, 3H). ^{13}C NMR (101 MHz, $CDCl_3$) δ 150.56, 138.84, 128.98, 121.47, 119.16, 115.41, 51.38, 51.30, 46.09, 45.93, 21.78, 13.00.

(*S*)-2-methyl-1-(*m*-tolyl)piperazine ((*S*)-58)

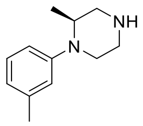

To a solution of **(*S*)-57** (150 mg, 0.52 mmol, 1.00 eq.) in DCM (4 mL) was added 1 mL TFA and the mixture was stirred for 2 h at room temperature. The reaction progress was monitored by TLC analysis. Upon full conversion of the starting materials, the reaction mixture was diluted with DCM (10 mL), washed with saturated $NaHCO_3$ solution, dried ($MgSO_4$), filtered and concentrated under reduced pressure. The residue was purified by silica gel column chromatography (DCM/MeOH,10/1) to give the product (90 mg, 0.47 mmol, 92%). ^{1}H NMR (400 MHz, $CDCl_3$) δ 7.14 (t, *J* = 7.5 Hz, 1H), 6.78 – 6.62 (m, 3H), 3.73 (qq, *J* = 6.7, 3.7, 3.3 Hz, 1H), 3.15 – 2.78 (m, 6H), 2.31 (s, 3H), 1.03 (d, *J* = 6.5 Hz, 3H). ^{13}C NMR (101 MHz, $CDCl_3$) δ 150.78, 138.76, 128.94, 120.80, 118.44, 114.69, 51.85, 51.40, 46.46, 45.97, 21.83, 12.64.

Ethyl 2-((4-((*R*)-3-methyl-4-(*m*-tolyl)piperazine-1-carbonyl)-2-nitrophenyl)sulfinyl)acetate ((*R*)-1)

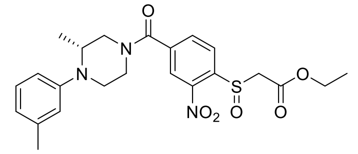

To a mixture of **(R)-58** (57.4 mg, 0.30 mmol, 1 eq.), **55** (100 mg, 0.33 mmol, 1.1 eq.) in DCM (5 mL) was added HATU (140 mg, 0.45 mmol, 1.5 eq.) and DiPEA (117 mg, 0.91 mmol, 3 eq.). Then the mixture was stirred overnight. The reaction progress was monitored by TLC analysis. Upon full conversion of the starting materials, the reaction mixture was diluted with DCM (10 mL), washed with water, dried ($MgSO_4$), filtered and concentrated under reduced pressure. The residue was purified by silica gel column chromatography (DCM/MeOH,10/1) to give the product (98 mg, 0.21 mmol, 69%). ^{1}H NMR (850 MHz, $CDCl_3$) δ 8.47 – 8.34 (m, 2H), 8.07 – 7.97 (m, 1H), 7.18 (t, *J* = 7.8 Hz, 1H), 6.83 – 6.68 (m, 3H), 4.47 – 4.08 (m, 4H), 3.78 (d, *J* = 13.8 Hz, 2H), 3.72 – 3.06 (m, 5H), 2.33 (s, 3H), 1.28 (td, *J* = 7.1, 1.7 Hz, 3H), 1.12 – 0.92 (m, 3H). ^{13}C NMR (214 MHz, $CDCl_3$) δ 166.88, 164.60, 149.51, 144.89, 143.86, 139.88, 139.17, 133.69, 129.20, 127.89, 124.24, 121.93, 119.36, 115.60, 62.44, 60.04, 52.25, 47.72, 45.62, 42.59, 21.79, 14.18, 12.71.

Ethyl 2-((4-((*S*)-3-methyl-4-(*m*-tolyl)piperazine-1-carbonyl)-2-nitrophenyl)sulfinyl)acetate ((*S*)-1)

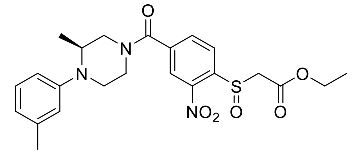

To a mixture of **(*S*)-58** (57.4 mg, 0.30 mmol, 1 eq.), **55** (100 mg, 0.33 mmol, 1.1 eq.) in DCM (5 mL) was added HATU (140 mg, 0.45 mmol, 1.5 eq.) and DiPEA (117 mg, 0.91 mmol, 3 eq.). Then the mixture was stirred overnight. The reaction progress was monitored by TLC analysis. Upon full conversion of the starting materials, the reaction mixture was diluted with DCM (10 mL), washed with water, dried ($MgSO_4$), filtered and concentrated under reduced pressure. The residue was purified by silica gel column chromatography (DCM/MeOH,10/1) to give the product (140 mg, 0.30 mmol, 98%).

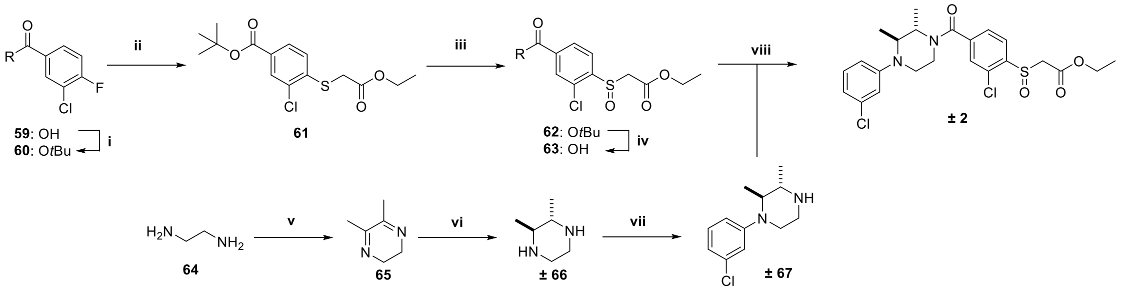

Scheme 2. Synthesis route for compound **2**. Reagents and conditions: i) Boc_2O, DMAP, *t*-BuOH, 65 °C. ii) Ethyl 2-

mercaptoacetate, K_2CO_3, ACN. iii) Oxone, MeOH / H_2O. iv) TFA, DCM. v) Diacetyl, Et_2O. vi) Na, EtOH, 80 °C. vii) 1-Bromo-3-chlorobenzene, KHMDS, 1,4-dioxane. viii) HATU, DiPEA, DCM.

***tert*-Butyl 3-chloro-4-fluorobenzoate (60)**

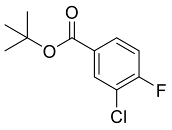

To a solution of 4-fluoro-3-chlorobenzoic acid (1 g, 5.73 mmol, 1 eq.) in *tert*-butanol (50 mL) was added Boc_2O (3.13 g, 14.3 mmol, 2.5 eq.) and DMAP (210 mg, 1.72 mmol, 0.30 eq.). Then the reaction mixture was stirred 65 °C overnight. The reaction progress was monitored by TLC analysis. Upon full conversion of the starting materials, the solvent was removed under reduced pressure and the residue was purified by silica gel column chromatography (Pentane/EA,100/1) to give the product (1.16 g, 5.04 mmol, 88%). ^{1}H NMR (500 MHz, $CDCl_3$) δ 8.03 (dd, *J* = 7.2, 2.2 Hz, 1H), 7.88 (ddd, *J* = 8.6, 4.7, 2.2 Hz, 1H), 7.17 (t, *J* = 8.6 Hz, 1H), 1.59 (s, 9H). ^{13}C NMR (126 MHz, $CDCl_3$) δ 163.9, 160.9 (d, *J* = 255.2 Hz), 132.3, 129.9 (d, *J* = 8.4 Hz), 129.3 (d, *J* = 3.6 Hz), 121.3 (d, *J* = 18.2 Hz), 116.5 (d, *J* = 21.6 Hz), 82.1, 28.3.

***tert*-Butyl 3-chloro-4-((2-ethoxy-2-oxoethyl)thio)benzoate (61)**

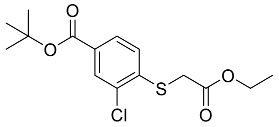

To a solution of **60** (1.16 g, 5.04 mmol, 1eq.) in ACN (10 mL) was added ethyl 2-mercaptoacetate (1.21 g, 10.1 mmol, 2 eq.) and K_2CO_3 (2.08g, 15.1 mmol, 3 eq.). Then the mixture was stirred overnight. The reaction progress was monitored by TLC analysis. Upon full conversion of the starting materials, the reaction mixture was diluted with DCM (50 mL), washed with water, dried ($MgSO_4$), filtered and concentrated under reduced pressure. The residue was purified by silica gel column chromatography (Pentane/Diethyl ether,10/1) to give the product (1.62 g, 4.60 mmol, 91%). ^{1}H NMR (400 MHz, $CDCl_3$) δ 7.94 (d, *J* = 1.8 Hz, 1H), 7.83 (dd, *J* = 8.3, 1.8 Hz, 1H), 7.32 (d, *J* = 8.3 Hz, 1H), 4.20 (q, *J* = 7.1 Hz, 2H), 3.74 (s, 2H), 1.58 (s, 9H), 1.26 (t, *J* = 7.1 Hz, 3H). ^{13}C NMR (101 MHz, $CDCl_3$) δ 168.8, 164.4, 140.6, 132.2, 130.6, 130.5, 128.2, 126.6, 81.8, 62.2, 34.5, 28.3, 14.2.

***tert*-Butyl 3-chloro-4-((2-ethoxy-2-oxoethyl)sulfinyl)benzoate (62)**

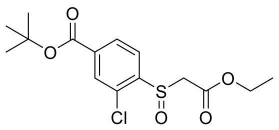

To a cooled solution of **61** (140 mg, 0.42 mmol, 1.00 eq.) in methanol (10mL) was dropwise added a solution of Oxone (26 mg, 0.42 mmol, 1.00 eq.) in water (2 mL) and the reaction mixture was stirred at rt for 2.5 h. The

reaction progress was monitored by TLC analysis. Upon full conversion of the starting materials, the mixture was diluted with water and extracted with DCM. The combined organic layers were washed with brine, dried ($MgSO_4$), filtered and concentrated under reduced pressure. The residue was purified by silica gel column chromatography (Pentane/Diethyl ether, 5/1) to afford the product (147 mg, 0.42 mmol, quant.). ^{1}H NMR (400 MHz, $CDCl_3$) δ 8.12 (d, J = 8.1 Hz, 1H), 8.06 – 7.92 (m, 2H), 4.29 – 4.18 (m, 2H), 4.03 (d, J = 13.1 Hz, 1H), 3.69 (d, J = 13.8 Hz, 1H), 1.61 (s, 9H), 1.26 (t, J = 7.0 Hz, 3H). ^{13}C NMR (126 MHz, $CDCl_3$) δ = 164.5, 163.6, 145.2, 136.5, 130.8, 130.1, 128.8, 126.5, 82.7, 62.4, 58.1, 28.2, 14.2.

3-Chloro-4-((2-ethoxy-2-oxoethyl)sulfinyl)benzoic acid (63)

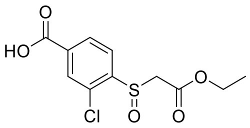

To a solution of **62** (1.39 g, 4.00 mmol, 1 eq.) in DCM (16 mL) was added 4 mL TFA and the reaction mixture was stirred for 6 h. The reaction progress was monitored by TLC analysis. Upon full conversion of the starting materials, the solvent was removed under reduced pressure and the residue was purified by silica gel column chromatography (DCM/MeOH, 10/1) to afford the product (1.04 g, 3.59 mmol, 90%). ^{1}H NMR (500 MHz, $CDCl_3$) δ 8.17 (dd, J = 8.1, 1.5 Hz, 1H), 8.07 (d, J = 1.5, 1H), 7.99 (d, J = 8.1, 1H), 4.26 – 4.14 (m, 2H), 4.04 (d, J = 14.0 Hz, 1H), 3.72 (d, J = 14.0 Hz, 1H), 1.23 (t, J = 7.1 Hz, 3H). ^{13}C NMR (126 MHz, $CDCl_3$) δ 166.5, 164.5, 145.4, 135.0, 131.2, 130.2, 129.3, 126.7, 62.5, 58.0, 14.1.

5,6-Dimethyl-2,3-dihydropyrazine (65)

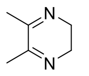

To a cooled (0°C) solution of ethylenediamine (6.6 mL, 100 mmol) in Et_2O (250 mL) was dropwise added a solution of 2,3-butanedione (8.8 mL, 100 mmol) in Et_2O (250 mL) and the suspension was allowed to stir for 16 h. The resulting clear liquid was dried using potassium hydroxide for 30 min. After filtration, the mixture was concentrated and the residue was purified by short-neck distillation which yielded the product (9.36 g, 85 mmol, 85%). ^{1}H NMR (400 MHz, $CDCl_3$) δ 3.36 (s, 4H), 2.15 (s, 6H). ^{13}C NMR (101 MHz, $CDCl_3$) δ 159.5, 44.9, 23.4.

(±) *trans*-2,3-Dimethylpiperazine (± 66)

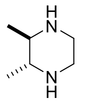

To a solution of **65** (9.36 g, 85 mmol, 1 eq.) in absolute ethanol (300 mL) was portion wise added sodium metal (23 g, 1 mol, 11.8 eq.) over six hours, after which the solution was refluxed for an additional 16 h. The slurry was neutralized by addition of acetic acid (50 mL) at 0 °C. The suspension was diluted with DCM, after stirring for 30 min the precipitated sodium acetate was filtered off. The filtrate was concentrated under reduced pressure and the residue was purified by silica gel column chromatography (Et_2O: MeOH: NH_4OH, 10:4:1) to afford the product (3.71 g, 32.5 mmol, 38%). ^{1}H NMR (500 MHz, $CDCl_3$) δ 3.90 (s, 1H), 2.98 (m, 4H), 2.53 (m, 2H), 1.12-1.09 (m, 6H).

^{13}C NMR (126 MHz, $CDCl_3$) δ 57.2, 45.8, 18.5.

(±) *trans*-1-(3-Chlorophenyl)-2,3-dimethylpiperazine (± 67)

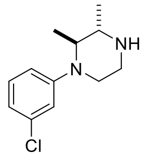

To a solution of **± 66** (0.70 g, 6.1 mmol, 1 eq.) in anhydrous dioxane (17 ml) were added 1-bromo-2-chlorobenzene (0.60 ml, 6.1 mmol, 1 eq.) and KHMDS solution (1M in THF, 6.1 ml, 6.1 mmol, 1 eq.). The reaction mixture was stirred at RT for 2 h. The reaction progress was monitored by TLC analysis. Upon full conversion of the starting materials, the mixture was diluted with DCM, washed with water, dried ($MgSO_4$), filtered and concentrated under reduced pressure. The crude product was purified using column chromatography (1% -> 10% MeOH in DCM with 1% TEA) to yield the product (0.51 mg, 2.0 mmol, 32%). ^1H NMR (400 MHz, $CDCl_3$) δ 7.20 (t, *J* = 8.1 Hz, 1H), 6.97 (t, *J* = 2.2 Hz, 1H), 6.89 (dddd, *J* = 18.0, 8.3, 2.1, 0.9 Hz, 2H), 3.24 – 2.82 (m, 6H), 1.31 (d, *J* = 6.6 Hz, 3H), 1.05 (d, *J* = 6.4 Hz, 3H). ^{13}C NMR (400 MHz, $CDCl_3$) δ153.01, 134.81, 130.07, 121.22, 120.23, 118.30, 57.84, 54.59, 48.88, 42.59, 19.04, 14.91

(±) Ethyl 2-((2-chloro-4-(4-(3-chlorophenyl)-*trans*-2,3-dimethylpiperazine-1-carbonyl)phenyl)sulfinyl)acetate (± 2)

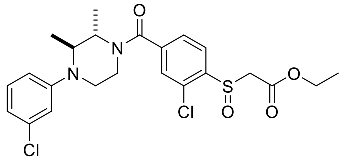

To a suspension of **63** (30 mg, 0.10 mmol, 1 eq.) in DCM (3 mL) was added HATU (39.2 mg, 0.10 mmol, 1.00 eq.) and DiPEA (31 mg, 0.30 mmol, 3 eq.). The mixture was stirred for 1 h. Then **± 66** (23.2 mg, 0.10 µmol, 1 eq.) was added and reaction mixture was stirred overnight. The reaction progress was monitored by TLC analysis. Upon full conversion of the starting materials, the mixture was diluted with DCM, washed with water, dried ($MgSO_4$), filtered and concentrated under reduced pressure. The crude product was purified using prep-HPLC to yield the product (38.3 mg, 77.3 µmol, 75 %). ^1H NMR (500 MHz, $CDCl_3$) δ 8.01 (d, *J* = 8.0 Hz, 1H), 7.54 (dd, *J* = 8.0, 1.6 Hz, 1H), 7.51 – 7.42 (m, 1H), 7.16 (t, *J* = 8.0 Hz, 1H), 6.83 – 6.78 (m, 2H), 6.70 (d, *J* = 8.4 Hz, 1H), 4.80 (t, *J* = 6.7 Hz, 1H), 4.62 (s, 1H), 4.29 – 4.17 (m, 2H), 4.04 (dd, *J* = 14.1, 1.7 Hz, 1H), 3.87 (d, *J* = 7.0 Hz, 1H), 3.69 (dd, *J* = 14.0, 1.2 Hz, 1H), 3.67 – 3.60 (m, 1H), 3.57 – 3.49 (m, 1H), 3.37 – 3.06 (m, 3H), 1.52 – 1.44 (m, 3H), 1.27 (t, *J* = 7.1 Hz, 3H), 1.16 – 097 (m, 3H). ^{13}C NMR (126 MHz, $CDCl_3$) δ 168.8, 168.3, 164.5, 151.3, 142.4, 140.5, 135.3, 130.8, 130.4, 128.4, 127.7, 127.1, 126.3, 125.8, 119.5, 116.3, 114.3, 62.5, 58.4, 56.2, 55.6, 49.8, 42.4, 41.3, 40.5, 36.6, 17.8, 16.8, 14.2, 12.8, 12.6. HRMS: Calculated for $[C_{23}H_{27}Cl_2N_2O_4S + H]^+$ = 497.1063, found = 497.1065.

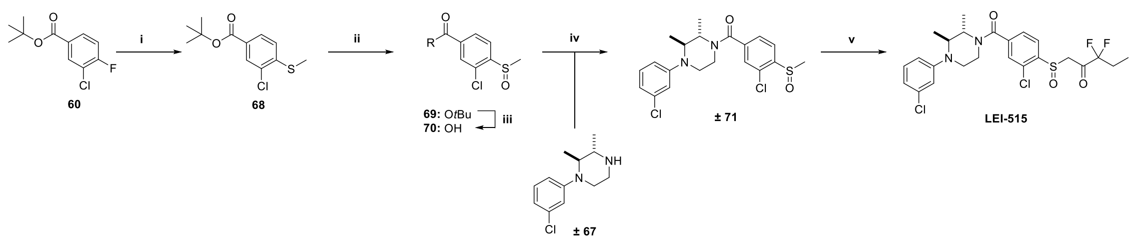

Scheme 3. Synthesis route for LEI-515. Reagents and conditions: i) NaSMe, K_2CO_3, ACN. ii) Oxone, MeOH / H_2O. iii) TFA, DCM. iv) EDCI, HOB, DiPEA, DCM. v) Ethyl 2,2-difluorobutanoate, LDA, THF, -78 °C.

***tert*-Butyl 3-chloro-4-(methylthio)benzoate (68)**

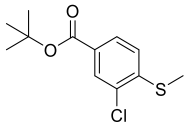

To a solution of *tert*-butyl 3-chloro-4-fluorobenzoate (0.51 g, 2.22 mmol, 1 eq.) in degassed DMF was added sodium methanethiolate (0.23 g, 3.32 mmol, 1.5 eq.) at -10 °C and the mixture was stirred at RT overnight. The reaction progress was monitored by TLC. Once completed, the mixture was diluted with Et_2O, washed with water, dried over anhydrous $MgSO_4$ and concentrated under reduced pressure. The residue was purified using column chromatography (Et_2O / pentane, 10%) to yield the product (0.24 g, 0.91 mmol, 41%). ^{1}H NMR (400 MHz, $CDCl_3$) δ 7.91 (d, *J* = 1.8 Hz, 1H), 7.85 (dd, *J* = 8.3, 1.8 Hz, 1H), 7.13 (d, *J* = 8.4 Hz, 1H), 2.49 (s, 3H), 1.59 (s, 9H). ^{13}C NMR (400 MHz, $CDCl_3$)δ 164.54, 143.80, 130.83, 129.94, 129.04, 128.05, 123.94, 81.49, 28.22, 14.92.

***tert*-Butyl 3-chloro-4-(methylsulfinyl)benzoate (69)**

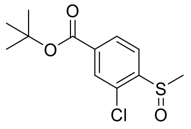

To a cooled (0 °C) solution of **68** (0.24 g, 0.91 mmol, 1 eq.) in MeOH was dropwise added an Oxone (0.50 g, 0.82 mmol, 0.9 eq.) in water and the mixture was stirred at RT for 2h. The reaction progress was monitored by TLC analysis. Upon full conversion of the starting materials, the mixture was diluted with EtOAc and washed with water. The organic layer was dried ($MgSO_4$), filtered and concentrated under reduced pressure. The residue was purified by silica column chromatography (pentane/EtOAc, 5/1) to yield the product (0.29 g, 1.17 mmol, quant.). ^{1}H NMR (500 MHz, $CDCl_3$) δ 8.13 (dd, *J* = 8.0, 1.6 Hz, 1H), 8.03 – 7.98 (m, 2H), 2.86 (s, 3H), 1.62 (s, 9H). ^{13}C NMR (500 MHz, $CDCl_3$) δ 163.34, 147.97, 135.70, 130.48, 129.52, 128.73, 125.20, 82.24, 41.37, 27.94.

3-Chloro-4-(methylsulfinyl)benzoic acid (70)

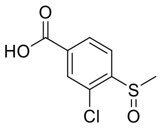

To a solution of **69** (0.26 g, 0.94 mmol) in DCM (8 mL) was added TFA (2 mL) and the reaction mixture was stirred

for 6.5 h. The reaction progress was monitored by TLC analysis. Upon full conversion of the starting materials, the solvent was removed and the residue was purified by sillica column chromatography (DCM/MeOH, 10/1) to yield the product (0.19 g, 0.87 mmol, 92%). ^{1}H NMR (400 MHz, MeOD) δ 8.22 (dd, *J* = 8.2, 1.6 Hz, 1H), 8.08 (d, *J* = 1.6 Hz, 1H), 7.98 (d, *J* = 8.1 Hz, 1H), 2.90 (s, 3H). ^{13}C NMR (400 MHz, MeOD) δ 167.19, 148.93, 136.49, 132.03, 131.20, 130.45, 126.47, 41.68.

(±) (3-Chloro-4-(methylsulfinyl)phenyl)(4-(3-chlorophenyl)-trans-2,3-dimethylpiperazin-1-yl)methanone (± 70)

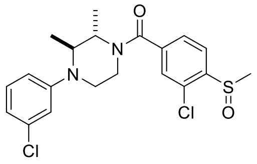

To a stirred suspension of 3-chloro-4-((2-ethoxy-2-oxoethyl)sulfinyl)benzoic acid (0.19 g, 0.87 mmol, 1 eq.) in DCM (10 mL) were added **± 67** (0.23 g, 1.04 mmol, 1.2 eq.), DIPEA (0.34 mg, 2.59 mmol, 3 eq.), HOBt (0.18 mg, 1.30 mmol, 1.5 eq.) and EDCl (0.25 mg, 1.30 mmol, 1.5 eq.). The mixture was stirred overnight. The reaction progress was monitored by TLC analysis. Once completed, the mixture was washed with water and brine, dried over anhydrous $MgSO_4$, filtered and concentrated. The crude product was purified using column chromatography (EtOAc / pentane, 60 %) to yield the product (0.27 g, 0.64 mmol, 74%). ^{1}H NMR (400 MHz, $CDCl_3$) δ 8.04 – 7.97 (m, 2H), 7.56 – 7.38 (m, 2H), 7.14 (t, *J* = 8.2 Hz, 1H).6.60 (d, *J* = 2.4 Hz, 1H), 6.56 – 6.47 (m, 1H), 4.85 – 4.49 (m, 1H), 3.98 – 3.07 (m, 5H), 2.82 (s, 3H), 1.42 – 1.33 (m, 3H), 1.28 – 1.06 (m, 3H). ^{13}C NMR (126 MHz, $CDCl_3$) δ 168.83, 151.33, 145.25, 140.18, 135.19, 130.47, 130.33, 128.43, 127.77, 125.85, 119.34, 116.13, 114.19, 56.08, 49.57, 42.29, 41.64, 36.46, 17.76, 12.52.

(±) 1-((2-Chloro-4-(4-(3-chlorophenyl)-*trans*-2,3-dimethylpiperazine-1- carbonyl)phenyl)sulfinyl)-3,3-difluoropentan-2-one (LEI-515)

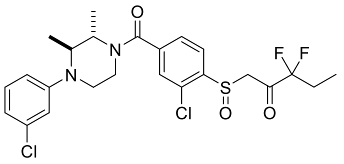

To a solution of **± 70** (20 mg, 0.05 mmol, 1 eq.) in THF (5 mL) was added LDA (0.025 mL, 2 M in THF, 0.05 mmol, 1 eq.) at -78 °C and the mixture was stirred for 10 min. Then ethyl 2,2-difluorobutanoate (72 mg, 0.47 mmol, 10 eq.) was added and the reaction mixture was stirred for 2 h at RT. The mixture was diluted with DCM (20 mL) and washed with water and brine, dried over anhydrous $MgSO_4$, filtered and concentrated. The crude product was purified using prep-HPLC to yield the product (2.5 mg, 0.05 mmol, 10%). ^{1}H NMR (500 MHz, $CDCl_3$) δ 8.04 (dd, *J* = 8.1, 4.5 Hz, 1H), 7.62 – 7.49 (m, 2H), 7.22 (t, *J* = 8.3 Hz, 1H), 6.89 (d, *J* = 7.2 Hz, 2H), 6.81 (d, *J* = 8.4 Hz, 1H), 4.41 (dd, *J* = 15.1, 4.3 Hz, 1H), 4.05 (dd, *J* = 15.2, 3.4 Hz, 1H), 3.85 – 3.13 (m, 8H), 2.17 – 2.02 (m, 3H), 1.54 (d, J = 6.8 Hz, 3H), 1.08 (t, *J* = 7.5 Hz, 3H). ^{13}C NMR (101 MHz, $CDCl_3$) δ 168.65, 168.17, 151.36, 142. 26, 140.93,

135.33, 130.65, 130.40, 128.67, 127.08, 126.51, 119.55, 118.14 (t, J = 253 Hz), 116.30, 114.28, 60.15, 56.22, 49.70, 40.52, 36.57, 25.52 (t, J = 23 Hz), 17.86, 12.60, 5.47 (t, J = 5 Hz). HRMS: Calculated for [$C_{24}H_{26}Cl_2F_2N_2O_3S$ + H_2O + H]+ = 549.1188, found = 549.1183.

Uncropped gels S3

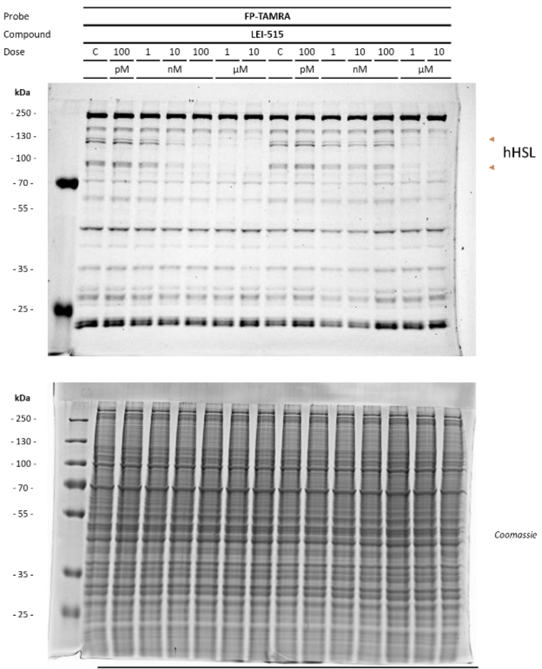

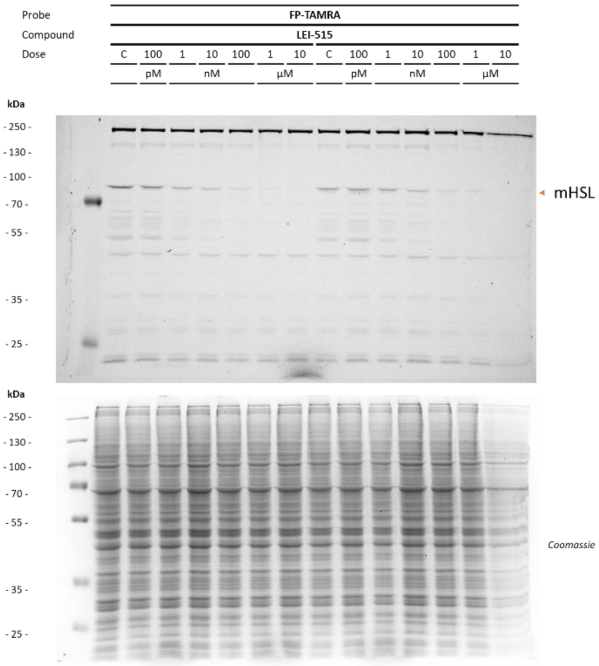

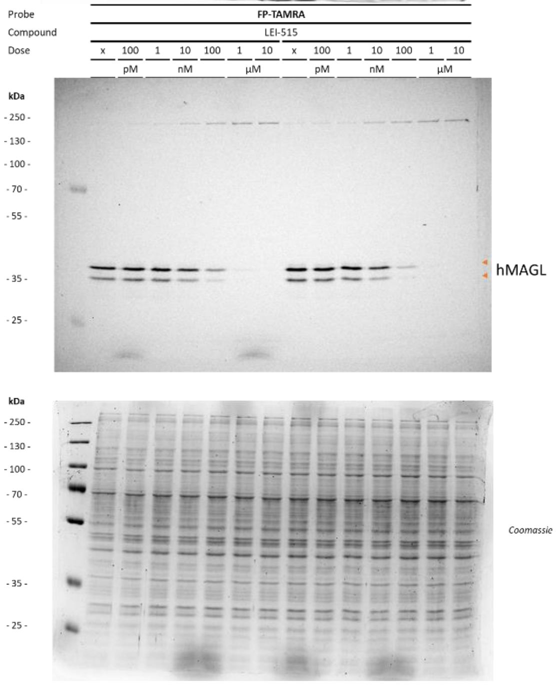

Uncropped gel and blots S4

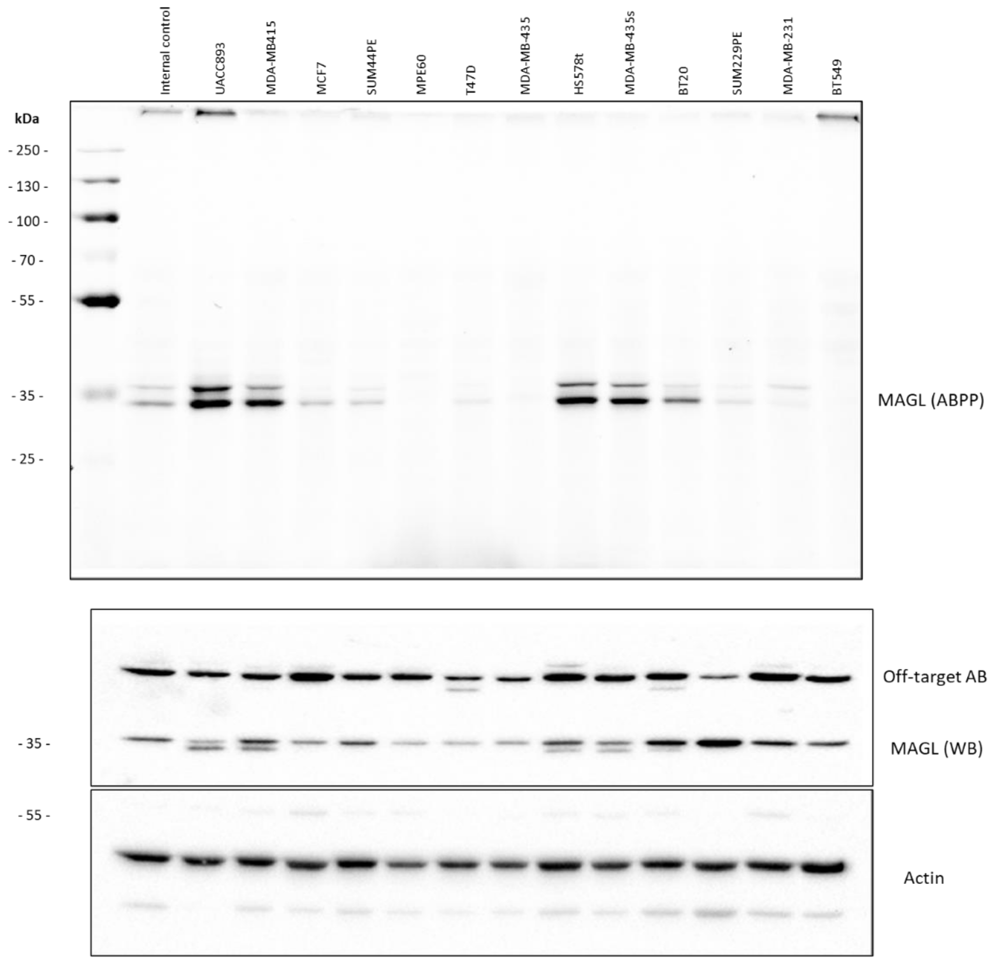

Supplement: Supplementary file 1 — Supplementary Information [file 41467_2023_43606_MOESM1_ESM.pdf]
