# Supplementary material for: A monoacylglycerol lipase inhibitor showing therapeutic efficacy in mice without central side effects or dependence
Source: Nat Commun. 2023 Dec 5;14:8039. doi: 10.1038/s41467-023-43606-3 (PMC10698032; doi:10.1038/s41467-023-43606-3)
Supplement: Supplementary file 3 — Reporting Summary [file 41467_2023_43606_MOESM3_ESM.pdf]

nature portfolio

Corresponding author(s): Prof. M. van der Stelt, Prof. A. G. Hohmann

Last updated by author(s): Sep 11, 2023

Reporting Summary

Nature Portfolio wishes to improve the reproducibility of the work that we publish. This form provides structure for consistency and transparency in reporting. For further information on Nature Portfolio policies, see our Editorial Policies and the Editorial Policy Checklist.

Statistics

For all statistical analyses, confirm that the following items are present in the figure legend, table legend, main text, or Methods section.

n/a	Confirmed	
☐	☒	The exact sample size (*n*) for each experimental group/condition, given as a discrete number and unit of measurement
☐	☒	A statement on whether measurements were taken from distinct samples or whether the same sample was measured repeatedly
☐	☒	The statistical test(s) used AND whether they are one- or two-sided<br>*Only common tests should be described solely by name; describe more complex techniques in the Methods section.*
☐	☒	A description of all covariates tested
☐	☒	A description of any assumptions or corrections, such as tests of normality and adjustment for multiple comparisons
☐	☒	A full description of the statistical parameters including central tendency (e.g. means) or other basic estimates (e.g. regression coefficient) AND variation (e.g. standard deviation) or associated estimates of uncertainty (e.g. confidence intervals)
☐	☒	For null hypothesis testing, the test statistic (e.g. *F*, *t*, *r*) with confidence intervals, effect sizes, degrees of freedom and *P* value noted<br>*Give P values as exact values whenever suitable.*
☒	☐	For Bayesian analysis, information on the choice of priors and Markov chain Monte Carlo settings
☒	☐	For hierarchical and complex designs, identification of the appropriate level for tests and full reporting of outcomes
☒	☐	Estimates of effect sizes (e.g. Cohen's *d*, Pearson's *r*), indicating how they were calculated

Our web collection on statistics for biologists contains articles on many of the points above.

Software and code

Policy information about availability of computer code

Data collection	XDS (version Jan31, 2020), SADABS 2008/1 (Bruker), PHASER 2.8.3, CCP4 suite 7.1.018, COOT 0.9.6, PyMOL Molecular Graphics System (version 1.8, Schrödinger).
Data analysis	Excel 2016 (Microsoft), Image Lab 6/7 (Bio-Rad), MassLynx 4.1 software (Waters Corporation), MaxQuant 2.0, Phoenix WinNonlin v.6.4, ImageJ 1.53, Prism 5/7/8/9 (GraphPad), MestReNova 9.1 (Mestrelab Research).

For manuscripts utilizing custom algorithms or software that are central to the research but not yet described in published literature, software must be made available to editors and reviewers. We strongly encourage code deposition in a community repository (e.g. GitHub). See the Nature Portfolio guidelines for submitting code & software for further information.

Data

Policy information about availability of data

All manuscripts must include a data availability statement. This statement should provide the following information, where applicable:

- Accession codes, unique identifiers, or web links for publicly available datasets
- A description of any restrictions on data availability
- For clinical datasets or third party data, please ensure that the statement adheres to our policy

Full-length cDNA encoding human MAGL (GenBank ID: BC006230.2 [https://www.ncbi.nlm.nih.gov/nuccore/BC006230.2]), mouse and human LIPE (referred to as 'HSL' elsewhere in manuscript; GenBank IDs: BC021642 [https://www.ncbi.nlm.nih.gov/nuccore/BC021642] and BC070041 [https://www.ncbi.nlm.nih.gov/nuccore/

March 2021

BC070041] respectively) are available at GenBank. The co-crystal structure of MAGL and LEI-515 is available in the PDB using code 8AQF [https://www.rcsb.org/structure/8AQF]. The mass spectrometry proteomics data generated have been deposited to the ProteomeXchange Consortium via the PRIDE partner repository with the dataset identifier PXD042220 [https://proteomecentral.proteomexchange.org/cgi/GetDataset?ID=PXD042220]. All other data needed to evaluate the conclusions in the paper are present in the paper, the Supplementary Materials and/or the source data file. Source data are provided with this paper.

Human research participants

Policy information about studies involving human research participants and Sex and Gender in Research.

Reporting on sex and gender	n/a
Population characteristics	n/a
Recruitment	n/a
Ethics oversight	n/a

Note that full information on the approval of the study protocol must also be provided in the manuscript.

Field-specific reporting

Please select the one below that is the best fit for your research. If you are not sure, read the appropriate sections before making your selection.

☒ Life sciences ☐ Behavioural & social sciences ☐ Ecological, evolutionary & environmental sciences

For a reference copy of the document with all sections, see nature.com/documents/nr-reporting-summary-flat.pdf

Life sciences study design

All studies must disclose on these points even when the disclosure is negative.

Sample size	No statistical methods were used to predetermine sample size. Sample sizes were chosen based on prior knowledge in the respective experiments and their intrinsic variability as performed in previous studies (Cao, Z. et al, Gastroenterology, 2013; Deng, L. et al. Biol Psychiatry, 2015; Den, L. et al Mol Pharmacol, 2015; van Esbroeck et al. Science 2017, Mock et al. Nat. Chem. Biol. 2020).
Data exclusions	No data were excluded.
Replication	Reproducibility of experiments was confirmed by the use of separately measured (biological) replicates and/or appropriate controls. All experiments were performed at least in three independent experiments, unless stated otherwise. Exceptions are the gel images in figure 1e and figure S1 due to limited quantity of the hits and dose response curves in figure 2b were measured in N=2 (individual plates), n=2 (technical replicates on same plate) or N=2, n=4 for controls. Attempts at replication were successful. The exact number of replicates per data point is indicated in figure legends.
Randomization	The animals were randomily distributed during the acute liver injury study, the chemotherapy induced neuropathic pain study and the CB1-dependent withdrawal study (figure 5-8). Randomization was not applicable for other experiments. All experiments were carried out with appropriate internal negative and/or positive controls as indicated.
Blinding	The investigators were blinded in the chemotherapy induced neuropathic pain study and the CB1-dependent withdrawal study (figure 6-8). In all other experiments investigators were not blinded because collection or analysis of the presented data was not prone to bias. These experiments are precise (and generally quantitative) measurements of enzyme activity, protein labeling and lipid levels and are not based on subjective assessments.

Reporting for specific materials, systems and methods

We require information from authors about some types of materials, experimental systems and methods used in many studies. Here, indicate whether each material, system or method listed is relevant to your study. If you are not sure if a list item applies to your research, read the appropriate section before selecting a response.

Materials & experimental systems

n/a	Involved in the study
☐	☒ Antibodies
☐	☒ Eukaryotic cell lines
☒	☐ Palaeontology and archaeology
☐	☒ Animals and other organisms
☒	☐ Clinical data
☒	☐ Dual use research of concern

Methods

n/a	Involved in the study
☒	☐ ChIP-seq
☒	☐ Flow cytometry
☒	☐ MRI-based neuroimaging

Antibodies

Antibodies used: Primary rabbit anti-MAGL (Abcam #ab24701, 1:200 dilution).

Validation: The used antibody was validated by commercial party Abcam. Statement Abcam: '' Validated in WB, IHC-P and tested in Rat samples. Cited in 30 publications.''

Eukaryotic cell lines

Policy information about cell lines and Sex and Gender in Research

Cell line source(s): HEK273T (catalogue number: CRL-3216), HS578T (catalogue number: HTB-126) and U87-MG (catalogue number: HTB-14) cells were obtained from ATCC. LLC-PK1 cells were obtained from Dr. A Schinkel, The Netherlands Cancer Institute.

Authentication: None of the cell lines were authenticated.

Mycoplasma contamination: All of the cell lines were negative for mycoplasma infection during all of our routine checks.

Commonly misidentified lines (See ICLAC register): None of the cell lines are present in the ICLAC register as commonly misidentified.

Animals and other research organisms

Policy information about studies involving animals; ARRIVE guidelines recommended for reporting animal research, and Sex and Gender in Research

Laboratory animals: C57BL/6J mice, 8 -12 weeks old.

Wild animals: This research did not involve wild animals.

Reporting on sex: In vivo target engagement studies were only conducted in male mice.
Acute liver injury was conducted in both male and female mice (7-9 per sex per group). Sex based analysis was performed, however no differences were found between male and female mice.
Age-matched male and female mice (7-8 per sex per group) were used to evaluate dose response, duration of action and effects of chronic dosing with LEI-515 in a mouse model of paclitaxel-induced peripheral neuropathy for each route of drug administration (oral, i.p.) (Fig 6a-f). Dose response was evaluated using a within subjects escalating dosing paradigm (figure 6a,d). Figure 6g-p shows data of only male mice (n=6-8 per group) as no major differences between the sexes were found in the data presented in figure 6a-f.

Field-collected samples: This research did not involve samples collected from the field.

Ethics oversight: All experimental procedures were approved by the Cantonal Veterinary Office Basel-Stadt; the National Institutes of Health, Institutional Animal Care and Use Committee of the National Institute on Alcohol Abuse and Alcoholism (Bethesda, Maryland); the Bloomington Institutional Animal Care and Use Committee of Indiana University and followed guidelines outlined by the International Association for the Study of Pain.

Note that full information on the approval of the study protocol must also be provided in the manuscript.
